# RRFERV stabilizes TEAD1 expression to mediate nasopharyngeal cancer radiation resistance rendering tumor cells vulnerable to ferroptosis

**DOI:** 10.1097/JS9.0000000000002099

**Published:** 2024-09-30

**Authors:** Qingqing Xu, Xin Wen, Chenglong Huang, Zaishan Lin, Zhen Xu, Ciming Sun, Li Li, Suixian Zhang, Shuanghong Song, Jiahao Lou, Zan Hou, Yuanyuan Chen, Xuan Li, Lei Chen

**Affiliations:** a State Key Laboratory of Oncology in South China, Guangdong Key Laboratory of Nasopharyngeal Carcinoma Diagnosis and Therapy, Guangdong Provincial Clinical Research Center for Cancer, Sun Yat-sen University Cancer Center, Guangzhou, China; b Department of Radiation Oncology, Sun Yat-sen University Cancer Center, Guangzhou, China; c United Laboratory of Frontier Radiotherapy Technology of Sun Yat-sen University & Chinese Academy of Sciences Ion Medical Technology Co, Guangzhou, China; d Department of Radiation Oncology, Nanfang Hospital, Southern Medical University, Guangzhou, China

**Keywords:** ferroptosis, nasopharyngeal carcinoma, radiation resistance, RRFERV, TEAD1

## Abstract

**Background:**

Long noncoding RNAs (lncRNAs) regulate various essential biological processes, including cell proliferation, differentiation, apoptosis, migration, and invasion. However, in nasopharyngeal carcinoma (NPC), the clinical significance and mechanisms of lncRNAs in malignant progression are unknown.

**Methods:**

RNA sequencing and bioinformatic analysis were used to determine the potential function of RRFERV (radiation-resistant but ferroptosis-vulnerable), and its biological effects were investigated using *in vitro* and *in vivo* experiments. Western blotting, quantitative real-time reverse transcription PCR, RNA immunoprecipitation (RIP) assays, and flow cytometry detected RRFERV expression. Ferroptosis and lipid peroxidation were added to evaluate the relationship between it and radiotherapy resistance.

**Results:**

LncRNA–RRFERV was both highly expressed in NPC tissues and radiation-resistant cells. RRFERV is associated with poor clinical outcomes of NPC patients and stabilizes TEAD1 by competitive binding with microRNA-615-5p and microRNA-1293. RRFERV–TEAD1 signaling axis leads to malignant progression and radiotherapy resistance of NPC. Furthermore, we observed that NPC radiotherapy-resistance cells exist in a fragile oxidative stress equilibrium, which makes them more sensitive to ferroptosis inducers. Surprisingly, we found that RRFERV–TEAD1 signaling axis also plays a key role in mediating the lipid peroxidation levels of NPC radiotherapy-resistance cells through transcriptional activation of ACSL4/TFRC.

**Conclusions:**

RRFERV serves as an independent prognostic factor in NPC. During the malignant progression of NPC caused by high expression of RRFERV, ferroptosis can be induced to effectively kill cancer cells and reverse the radiotherapy resistance of NPC cells, suggesting a potential treatment approach for recurrent and refractory NPC.

## Introduction

Highlights

- RRFERV promotes the progression and radiation resistance of nasopharyngeal cancer through the TEAD1 pathway.
- RRFERV stabilizes TEAD1 by competitively binding with microRNA-615-5p and microRNA-1293.
- Radiation-resistant cells are in a delicate balance between lipid peroxidation and increased vulnerability to ferroptosis.

Nasopharyngeal carcinoma (NPC) is a unique head and neck cancer that has a distinct geographical distribution and different biological characteristics from other head and neck tumors^1^. It is endemic in Southeast Asia and Southern China, where it accounted for over 70% of the estimated 133 354 worldwide cases in 2020^2^. Approximately 95% of cases in endemic areas are non-keratinizing NPC^3^, which is radiosensitive; therefore, radiotherapy (RT) is the primary treatment modality for nonmetastatic NPC. With advances in contemporary cancer management, including intensity-modulated radiotherapy and combined chemotherapy, the survival rate of patients with nonmetastatic NPC has increased greatly; however, after standard treatment, around 30% of patients experience metastasis and recurrence, representing the leading cause of death in patients with NPC^4,5^. These patients have developed tolerance to conventional radiotherapy and chemotherapy, and urgently need to find a new combination treatment regimen^6^.

Currently, tumor-node-metastasis (TNM) staging is mainly used to determine the treatment strategy for patients with NPC. However, there are significant differences in treatment response and survival outcomes (recurrence and/or metastasis) among patients with NPC at the same stage treated with the same treatment^4,7^, suggesting that TNM staging based on the extent of extrinsic tumor anatomical invasion cannot accurately predict patient prognosis and guide individualized treatment. Therefore, there is an urgent need to identify new biomarkers to further reflect the intrinsic biological heterogeneity of the tumor, which could be combined with TNM staging to screen those patients who have a high risk of treatment failure and thus require individualized treatment.

A class of non-protein-coding RNAs longer than 200 bp have been identified and named long noncoding RNAs (lncRNAs)^8^. LncRNAs can function as protein scaffolds, signaling molecules, and decoys and regulate various essential biological processes, including cell proliferation, differentiation, apoptosis, migration, and invasion^9–11^. In addition, lncRNAs can act as competing endogenous RNAs (ceRNAs) to sponge microRNAs (miRNAs) during transcriptional regulatory processes^12^. Studies have demonstrated that the abnormal expression of lncRNAs is associated with the pathogenesis of various tumors, including non-small cell lung cancer, ovarian cancer, prostate cancer, and colorectal cancer, and thus can be used as prognostic markers and therapeutic targets for tumors^13–16^. Meanwhile, the role of lncRNAs in NPC has been gradually revealed, for example, LOC401317, lncRNA-LET, and DANCR affect NPC cell proliferation, apoptosis, and metastasis, respectively^17–19^. Further investigation of the molecular mechanisms by how lncRNA affects the fate of tumor cells and the radiosensitivity of nasopharyngeal carcinoma is required.

Ferroptosis is a recently discovered programmed cell death pattern that is independent of the caspase family and does not involve apoptosis^20^. The important factors regulating the initiation of ferroptosis include Acyl-CoA synthetase long-chain family member 4 (ACSL4) and the transferrin receptor (TFRC)^21,22^. ACSLs mainly induce the peroxidation of polyunsaturated fatty acids on the cell membrane, and TFRC mainly affects the transport of Fe^3+^ ions. The suppressors of ferroptosis include glutathione peroxidase 4 (GPX4), which mainly affects the removal of glutathione peroxidation^23^, and solute carrier family 7 member 11 (SLC7A11), which mainly affects intracellular cystine transport^24^. They are the major brake proteins of the ferroptosis signaling pathway. A variety of stimuli in the intracellular or external environment can affect the occurrence of ferroptosis by affecting lipid peroxidation and the Fe^3+^ concentration. Traditionally, chemoradiotherapy and immunotherapy mainly mediate cell apoptosis. However, recent studies have shown that the mechanism of cell death induced by these conventional tumor therapies is very complicated, and ferroptosis plays an important role in this process. Classical oncogenes, such as RAS^25^, can regulate the occurrence of ferroptosis by affecting lipid peroxidation, and p53 can induce ferroptosis by inhibiting the transcription of SLC7A11^26,27^. Therefore, it can be seen that ferroptosis plays a key role in the regulation of carcinoma development. Inducing ferroptosis might be an effective way to kill tumors, and thus has good clinical application prospects^28,29^.

In this study, we provided compelling evidence that the expression of lncRNA RRFERV was significantly increased in NPC tissue and was related to poor prognosis in patients with NPC. We named this lncRNA RRFERV since it is radiation-resistant but ferroptosis-vulnerable. RRFERV is from the study by Liang *et al*. (GSE180272) and its official symbol is LINC01770 (Gene ID: 102724312). *In vivo* and *in vitro* assays demonstrated that RRFERV promoted NPC cell proliferation, migration, and invasion. Furthermore, RRFERV sponges miR-615-5p and miR-1293 and thus regulates TEA domain transcription factor 1 (TEAD1) expression and activates the Hippo signaling pathway. To further investigate the molecular mechanism of radioresistance in NPC, we constructed radioresistant cell lines. In addition, the RRFERV–TEAD1 signaling axis was found to mediate the radiation resistance of NPC. Moreover, RRFERV–TEAD1 signal axis could also promote the transcription of the key promotion genes of ferroptosis, ACSL4 and TFRC, leading to a fragile oxidative stress balance in radioresistant NPC cells, which is vulnerable to ferroptosis inducer. RT resistance occurs in the majority of patients with recurrent NPC, which is the critical reason for the failure of NPC treatment. Our findings revealed the clinical relevance of RRFERV and its function and mechanism associated with the malignant progression in NPC. In this process, the RRFERV–TEAD1 axis can not only promote the malignant progression of NPC but also make NPC cells more susceptible to ferroptosis, indicating a novel therapeutic strategy for individualized treatment.

## Materials and methods

### Clinical samples

All patients provided written informed consent. Relevant information about all patients is displayed in the supplemental materials (Supplementary Tables S3, S4, Supplemental Digital Content 1, http://links.lww.com/JS9/D498). For the microarray analysis, 18 pairs of freshly frozen NPC tissues and normal nasopharyngeal epithelial tissues were obtained by biopsy. For the prognostic analysis, 220 formalin-fixed NPC paraffin specimens were collected between January 2006 and December 2009. The following inclusion criteria were used: NPC that was confirmed pathologically, previously untreated, and nonmetastatic; patients did not have other previous malignancies or serious liver, kidney, heart, or lung disease; patients received radical radiotherapy, with or without platinum-based chemotherapy; with regular follow-up. The 8th edition of the American Joint Committee on Cancer (AJCC) guidelines were used to restage all the patients, and clinical characteristics and long-term follow-up data were collected.

### Cell culture

The human immortalized nasopharyngeal epithelial cell line (NP69) was cultured in keratinocyte serum/free medium (Invitrogen, Waltham, MA, USA) supplemented with bovine pituitary extract (BD Biosciences, San Jose, CA, USA) at 37°C under 5% CO_2_. Seven human NPC cell lines (SUNE-1, HONE-1, CNE-1, CNE-2, HNE1, HK-1, and 5-8F) were maintained in Roswell Park Memorial Institute (RPMI)1640 medium or Dulbecco’s modified Eagle’s medium (DMEM) (both Invitrogen) containing 10% fetal bovine serum (FBS; Gibco, Grand Island, NY, USA).

### RNA extraction and quantitative real-time reverse transcription PCR (qRT-PCR)

Total RNA from NPC cell lines and NPC paraffin specimens was extracted using TRIzol (Invitrogen) and reversed transcribed to cDNA using HiScript III RT SuperMix (Vazyme, Nanjing, China) according to the manufacturer’s instructions. The qPCR step of the qRT-PCR protocol was performed using Platinum SYBR Green qPCR SuperMix-UDG reagents (Vazyme) on the CFX96 Touch sequence detection system (Bio-Rad Laboratories Inc., Hercules, CA, USA). Primers for microRNA-615-5p, microRNA-1293, and U6 were obtained from GeneCopoeia (Rockville, MD, USA). The level of *GAPDH* mRNA (encoding gGlyceraldehyde-3-phosphate dehydrogenase) was employed for normalization and the 2^−ΔΔCT^ method^30^ was used to calculate the relative expression levels. Supplementary Table S1, Supplemental Digital Content 1, http://links.lww.com/JS9/D498 shows the specific primers used for qPCR.

### Oligonucleotide transfection and generation of stably transfected cell lines

The small interfering RNAs (siRNAs) targeting RRFERV and TEAD1, miR-615-5p mimics, miRNA-1293 mimics, the miR-615-5p inhibitor, the miRNA-1293 inhibitor, and the scrambled negative control siRNA (si-NC) were purchased from GenePharma (Shanghai, China) (Supplementary Table S2, Supplemental Digital Content 1, http://links.lww.com/JS9/D498). Plasmid vectors (pCDNA-RRFERV, shRRFERV, pcDNA-3.1-TEAD1, and empty vectors) for transfection were prepared by GeneCopoeia.

shRNA was transfected into NPC cells using RNAmate (Invitrogen) or Lipofectamine 3000 reagent (Invitrogen). Cells were harvested and assayed 48 h after transfection. shRRFERV was inserted into vector pLKO.1 and the psPAX2 packaging plasmid (Addgene, Watertown, MA, USA) and pMD2.G envelope plasmid (Addgene) were co-transfected into 293T cells using polyethyleneimine (PEI; Polysciences, Warrington, PA, USA). After transfection, the medium was changed at 12 h, the supernatant was collected at 48 h, and then the virus contents of the indicated shRNA were used for the infection of NPC cells. Puromycin (1 mg/ml) was used to screen for stably transfected cells and qRT-PCR was used to determine whether a stable knockdown or high expression NPC cell line of RRFERV was successfully established. Transfection with lentiviral plasmids overexpressing TEAD1 was carried out to gain NPC cells stably co-expressing shRNA-RRFERV and TEAD1.

### Microarray analysis, RNA sequencing, and bioinformatic analysis

An Arraystar LncRNA Microarray 3.0 V3.0 platform (Agilent Technologies, Santa Clara, CA, USA) was used to determine lncRNA expression in NPC and normal tissues. We isolated total RNA from SUNE-1 transiently transfected with shRRFERV, control shRNA, RRFERVOE (RRFERV overexpression), and control OERNA, purified, and subjected it to RNA sequencing (RNA-seq), carried out by BGI Genomics (Beijing, China). Differentially expressed genes were screened using a threshold of *P*<0.05 and fold change ≥1.5. The DAVID software was used to carry out Gene Ontology (GO) Enrichment Analysis and Kyoto Encyclopedia of Genes and Genomes (KEGG) pathway analysis of the differentially expressed genes (DEGs). In addition, we determined the biological functions enriched in NPC in response to RRFERV knockdown GO and gene set enrichment analysis (GSEA). Significant terms were selected based on a threshold of *P*<0.05. The RNA-seq data were deposited in the Gene Expression Omnibus (GEO) at the National Center for Biotechnology Information (accession number GSE236418).

### CCK-8 and colony formation assays

Cells at 1×10^3^ cells per well were seeded in triplicate in 96-well plates and their viability was measured daily for 7 days using a Cell Counting Kit-8 (CCK-8) assay (Dojindo, Kumamoto, Japan). In the assay, 10 μl of CCK-8 solution was added to each well. After 2 h of incubation, a spectrophotometric plate reader (ELX800, BioTek, Winooski, VT, USA) was used to measure the absorbance at 450 nm. To determine their colony formation ability, 500 cells were cultured in 2 ml of medium in a 6-well plate for 1 or 2 weeks. The formed cell colonies were washed using phosphate-buffered saline (PBS), fixed in paraformaldehyde, crystal violet stained, and counted using ImageJ software (NIH, Bethesda, MD, USA).

### Flow cytometry

Cells stained with propidium iodide (PI) or SYTOX Green (both Invitrogen) were analyzed by microscopy or flow cytometry to observe cell death. Cells were fixed with 70% ice-cold ethanol overnight and treated with RNase. Finally, cells were stained with propidium iodide (PI) for 30 min at 4°C and submitted for cell cycle analysis on flow cytometry (Ex=488 nm).

### Lipid peroxidation measurement

Cells at 1×10^5^ cells per well were seeded in 12-well plates. One day later, the indicated compounds were used to treat the cells, which were then collected, stained using 5 μM BODIPY 581/591 C11 (Invitrogen, D3861) for 30 min at 37°C, and then subjected to flow cytometry analysis. For BODIPY 581/591 C11 staining, the signals from both oxidized C11 (FITC channel) and non-oxidized C11 (PE channel) were monitored. The ratio of the mean fluorescent intensity (MFI) of FITC to the MFI of PE was calculated for each sample. The data were normalized to control samples to determine the relative lipid peroxidation. The experiments were carried out at least three times, with 5000 cells being analyzed in each group.

### In vitro assays of invasion and migration

Cell invasion and migration were determined using Transwell chambers (Corning Inc., Corning, NY, USA). Cells were cultured in 200 ml of medium without serum and then added to the upper chamber, which did or did not contain Matrigel (BD Biosciences); the lower chamber contained medium with 10% FBS. After incubation for 12 or 24 h, cells under the membrane were fixed and stained to observe their migration or invasion.

### RNA pull-down assay

Full-length sense and antisense sequences of RRFERV were transcribed *in vitro* using a MEGAscript T7 Transcription Kit (Thermo Fisher Scientific, Waltham, MA, USA). A Pierce RNA 30 End Desthiobiotinylation Kit (Thermo Fisher Scientific) was used to purify and biotinylate the resultant RNA. Cell under test were lysed and combined with the transcribed RNA for pull-down experiments, performed using a Pierce Magnetic RNA-Protein Pull-Down Kit (Thermo Fisher Scientific), followed by analysis using western blotting.

### Western blotting

NPC cell protein extracts were obtained and subjected to 10–14% sodium dodecyl sulfate-polyacrylamide gel electrophoresis (SDS-PAGE), followed by electrotransfer onto polyvinylidene fluoride membranes (Millipore, Billerica, MA, USA). Blocking was performed using 5% skim milk for 1 h, followed by overnight incubation with primary antibodies and then with horseradish peroxidase (HRP) conjugated secondary antibodies for 1 h. The primary antibodies were as follows: anti-ACSL4 (Abcam, Cambridge, MA, USA, 1:1000), anti-GPX4 (Abcam, 1:1000), anti-SLC7A11 (CST, Danvers, MA, USA, 1:500), anti-TEAD1 (CST, 1:500), and TFRC (also known as CD71; CST, 1:1000). Between incubations, three washes were performed using Tris-buffered saline-Tween20 (TBST) for 5 min each time. Immunoreactivity was detected using Clarity Western ECL substrate (Bio-Rad) followed by chemiluminescence detection using the ChemiDoc MP imaging system (Bio-Rad).

### RNA immunoprecipitation (RIP) assay

To determine if RRFERV could interact with or bind to argonaute 2 (AGO2)/TEAD1 in HONE-1 and SUNE-1 cells, we used an EZ-Magna RIP Kit (Millipore). NPC cell lysates were co-incubated with anti-TEAD1, anti-Ago2, or anti-IgG antibody-conjugated magnetic beads (68 h, 4°C). The magnetic beads were then collected and washed with RIP buffer. Finally, the beads were resuspended in the TRIzol regent to isolate the co-precipitated RNA for qRT-PCR analysis.

### Induction of ferroptosis

The compounds Erastin (E7781, Sigma-Aldrich) and RSL3 (HY-100218A, MCE, Monmouth Junction, NJ, USA) were used to induce ferroptosis in NPC cells, followed by flow cytometry and CCK-8 assays to detect cell viability.

### Fluorescence in-situ hydration (FISH) and immunofluorescence (IF)

Probes for RRFERV were synthesized by RiboBio (Guangzhou, China) for FISH detection of the co-localization and interaction of RRFERV and TEAD1. Cells were grown to 30% confluence and seeded on glass coverslips, with or without RSL3 treatment. The coverslips were washed briefly with PBS, fixed using 4% paraformaldehyde for 30 minutes at room temperature, blocked using 4% bovine serum albumin in PBS for 1 h, and then incubated with anti-TEAD1 antibodies overnight at 4°C. Alexa Fluor 647-labeled goat anti-rabbit IgG antibody was then incubated with the cells on the coverslips for 1 h at room temperature, stained for 5 min using 1 μg/ml 4′,6-diamidino-2-phenylindole (DAPI), rinsed using PBS and dried. An Olympus FV-1000 confocal microscope (Tokyo, Japan) was used to observe the cells (100× magnification).

### Models of in vivo tumor growth and metastasis

The Institutional Animal Care and Use Committee (approval number L025501202208011) approved all the animal procedures. Nude mice were purchased from Gempharmatech-GD (Nanjing, China). For the tumor growth model, RRFERV stable knockdown or overexpression NPC cells suspended in PBS were injected subcutaneously into the nude mice to establish a subcutaneous transplantation tumor model. When the tumors had grown to a palpable size, the mice were grouped randomly into four or six groups, as indicated in the figure legends, and injected intraperitoneally with sulfasalazine (SAS) (50 mg/kg); the mice were treated 6 Gy. On day 30, the mice were dissected to observe the size and weight of the tumors. To construct a tumor lung metastasis model, NPC cells were injected via the tail vein. To construct a model of lymph node metastasis in the popliteal fossa of the foot of nude mice, NPC cells were injected via their footpads. At the end of the experiment, excised tissues were fixed in paraffin and made into sections. The sections were then deparaffinized and rehydrated, and the antigen was retrieved. For IHC, the sections were incubated with primary antibodies recognizing ACSL4 (Abcam), GPX4(Abcam), SLC7A1(CST), TEAD1 (CST), and CD71(CST), followed by incubation with secondary antibodies. Finally, the sections were stained using diaminobenzidine (Sigma) and counterstained using hematoxylin (Sigma).

### Statistical analysis

Statistical analyses were performed using SPSS 23.0 (IBM Corp., Armonk, NY, USA) or GraphPad Prism (version 9.0, GraphPad Software, Inc., La Jolla, CA, USA). Each experiment was repeated at least three times and the data were expressed as the mean±SD. For quantitative variables, groups were compared using analysis of variance (ANOVA) or Student’s *t* test. For categorical variables, the chi-squared test or Fisher’s exact test was used to compare the groups. The Kaplan–Meier method was used to plot the survival curves, and the log-rank test was used for group comparisons. The effects of variables on survival outcomes were analyzed using univariate and multivariate Cox regression analysis. All tests were two-tailed and *P*<0.05 was considered statistically significant.

## Results

### RRFERV is upregulated in NPC and correlates with poor prognosis

**Figure 1:**
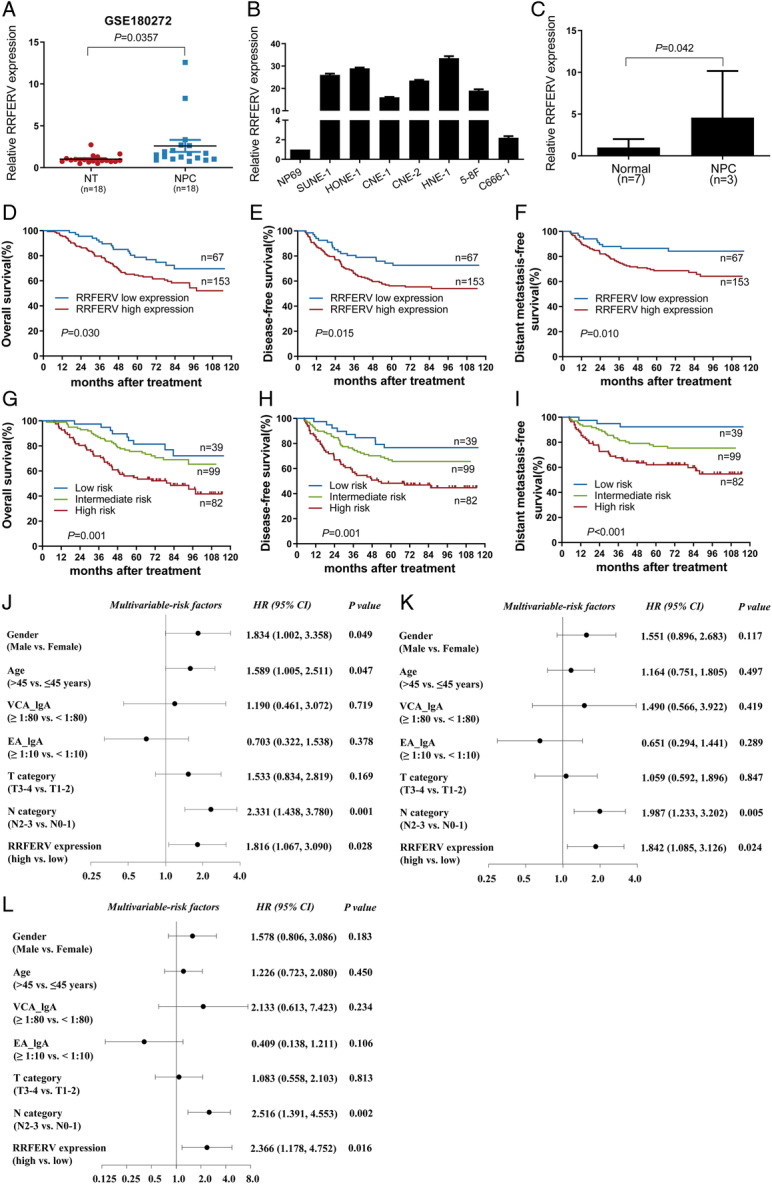
RRFERV is upregulated in NPC and correlates with poor prognosis. (A) Relative expression of RRFERV in NPC (*n*=18) and normal nasopharyngeal epithelial tissues (*n*=18). (B) Relative expression of RRFERV in NP69 and seven NPC cell lines. (C) RRFERV expression was determined by using qRT-PCR in NPC (*n*=13) tissues and normal nasopharynx tissues (*n*=7). (D–F) Kaplan–Meier curves showing overall survival (D), disease-free survival (E), and distant metastasis-free survival (F) associated with high or low expression of RRFERV. (G–I) Kaplan–Meier analysis of overall survival (G), disease-free survival (H), and distant metastasis-free survival (I) in a prognostic prediction model in patients in the low-risk group (low RRFERV expression and N0–1 stage, *n*=39), intermediate-risk group (low RRFERV expression and N2–3 stage or high RRFERV expression and N0–1 stage, *n*=99), and high-risk group (high RRFERV expression and N2–3 stage, *n*=82). (J–L) Multivariate Cox regression analysis of clinical prognostic parameters in 220 patients with NPC according to multivariate Cox regression analysis. Data are shown as the mean±SEM, *P*<0.01 (*n*=3 independent experiments).

Previously, based on the study by Liang *et al*. (GSE180272), we examined the expression of lncRNAs in 18 pairs of NPC and normal tissues using Arraystar LncRNA Microarray 3.0 microarrays and found significantly different expression in the two groups of samples, allowing us to distinguish tumor tissues from normal tissues with complete accuracy, with RRFERV expression being significantly higher in NPC tissues (Fig. 1A)^31^. In addition, we found that all seven NPC cell lines had higher RRFERV expression compared with that in the immortalized nasopharyngeal normal epithelial cell line NP69 (Fig. 1B). This suggested that RRFERV is involved in the development and progression of NPC. To validate the microarray results, we examined the expression of RRFERV using qRT-PCR, which showed significantly higher RRFERV expression in NPC tissues (Fig. 1C). To determine the relationship between the expression level of RRFERV and the prognosis of patients with NPC, we examined the expression of RRFERV in paraffin tissues of 220 NPC cases using qRT-PCR and performed survival analysis. The results revealed that patients with high RRFERV expression had a poorer overall survival (OS), disease-free survival (DFS), and distant metastasis-free survival (DMFS) than those with low RRFERV expression (all *P*<0.05; Fig. 1D–F). Moreover, a prognostic model based on RRFERV expression status and N stage was constructed. We divided the patients into three groups: the low-risk group (low RRFERV expression and N0–1 stage, *n*=39), the intermediate-risk group (low RRFERV expression and N2–3 stage or high RRFERV expression and N0–1 stage, *n*=99), and the high-risk group (high RRFERV expression and N2–3 stage, *n*=82) (all *P*<0.05; Fig. 1G–I). According to multivariate Cox regression analysis, TNM stage and RRFERV expression were independent factors affecting NPC patient prognosis (both *P*<0.05; Fig. 1J–L). Overall, our results showed that RRFERV expression combined with the N stage could be a promising indicator to guide the individualized treatment of patients. The above results tentatively suggested that RRFERV is associated with the prognosis of NPC and has potential as a promising molecular indicator to guide individualized patient treatment.

### RRFERV promotes NPC proliferation and metastasis

**Figure 2:**
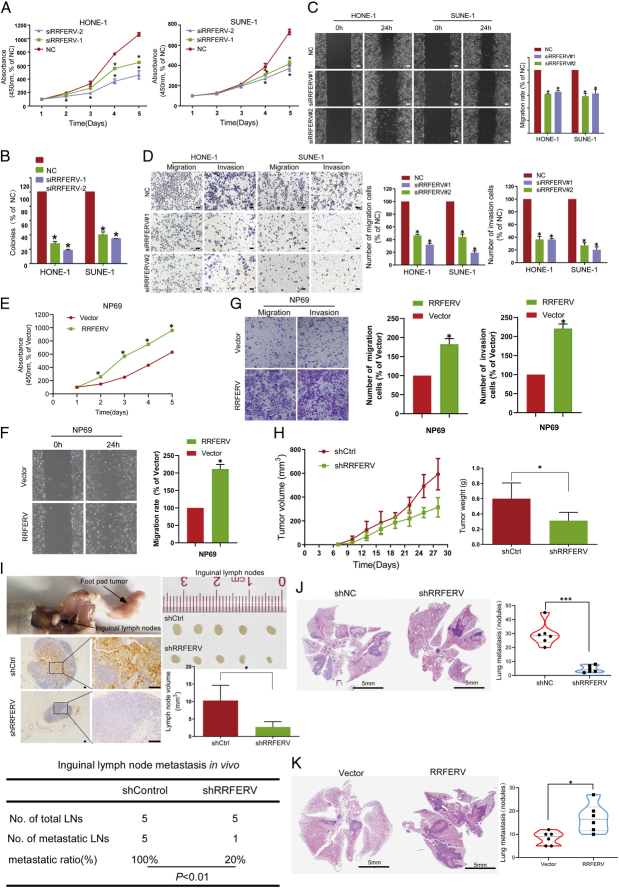
RRFERV promotes the metastasis and proliferation of NPC. (A) SiRRFERV and the scrambled control were transfected into HONE-1 and SUNE-1 cells, and cell proliferation was detected using a CCK-8 assay. (B) Colony formation assays of HONE-1 and SUNE-1 cells transfected with siRRFERV and the scrambled control. (C) Wound-healing assays of the above cells. (D) Transwell assays were performed to determine the migration and invasion abilities of cells transfected with siRRFERV or the scrambled control. (E–G) RRFERV overexpression and vector control plasmids were transfected in NPC cells, followed by CCK-8 assays (E), wound-healing assays (F), and transwell assays (G). Scale bar=500 μm. (H) A tumor growth model was established by transplanting SUNE-1 cells (silenced for RRFERV or not) into nude mice axillae. (I) Representative images of mouse footpads and inguinal lymph node metastasis models. (J) NPC cells were stably transfected with the virus of shRRFERV and shNC, and the cells were injected into the tail vein of nude mice, the lung metastasis nodes were observed by HE stains. (K) NPC cells were stably transfected with the virus of RRFERV overexpression and vector control, and the cells were injected into the tail vein of nude mice; the lung metastasis nodes were observed by HE stains.

Next, we characterized the carcinogenic phenotype in SUNE-1 and HONE-1 cells via RRFERV silencing CCK-8, and colony formation assays were performed, which revealed that NPC cell proliferation was reduced significantly after RRFERV silencing (Fig. 2A, B). In addition, wound-healing and transwell assays were performed, which showed that RRFERV silencing inhibited NPC cell migration and invasion (Fig. 2C, D, all *P*<0.01). CCK-8 and colony formation assays revealed NP69 transfected with RRFERV overexpression compared with those transfected with scrambled vectors promoted proliferation (Fig. 2E, all *P*<0.01). Moreover, wound-healing and transwell assays showed that RRFERV overexpression increased NP69 cell migration and invasion (Fig. 2F, G, all *P*<0.01). To further determine whether RRFERV upregulation could promote the progression of NPC, RRFERV was stably overexpressed in NPC cells via lentivirus transfection. Exogenous RRFERV overexpression increases NPC cell proliferation, migration, and invasion (Supplementary Fig. S1A–C, Supplemental Digital Content 2, http://links.lww.com/JS9/D499, all *P*<0.01).

Moreover, models of xenograft growth and inguinal lymph node metastasis were constructed to evaluate the effect of RRFERV on the growth and metastasis of NPC tumors *in vivo*. We subcutaneously injected SUNE-1 cells into nude mice and found that the growth rate of the shRRFERV group was significantly slower, and the tumor weight was significantly decreased compared with those in the scrambled control group (Fig. 2H). In addition, the volumes of the metastatic inguinal lymph nodes and the number of metastatic lung nodules in the RRFERV silenced group were reduced significantly compared with those in the control group (Fig. 2I, *P*<0.05). Furthermore, the RRFERV knockdown group showed fewer and smaller microscopic metastatic tumor nodules. To further investigate the effect of RRFERV on the malignant progression of NPC, SUNE-1 cells stably overexpressing RRFERV were assessed for their effect on tumor metastasis via injection into nude mice via tail veins. The number and size of lung metastases were significantly increased in the RRFERV overexpression group (Fig. 2J, K). We normalized the tumor volume when measuring the metastasis. We counted the ratio of inguinal lymph node volume/footpad tumor volume of each nude mouse, and the results showed that the ratio in the RRFERV knockdown group was lower than that in the ctrl group, which proved that knockdown of RRFERV could inhibit tumor metastasis. Meanwhile, we detected the EMT-related indexes by western blotting after the knockdown of RRFERV in NPC cells, and the analysis results showed that the RRFERV knockdown significantly upregulated the level of E-cadherin, but downregulated the level of N-cadherin (Supplementary Fig. S1D, E, Supplemental Digital Content 2, http://links.lww.com/JS9/D499, all *P*<0.01). These above results suggested that RRFERV might promote tumor formation and development by affecting the proliferation, migration, and invasion abilities of NPC cells.

### RRFERV promotes NPC progression by regulating TEAD1

**Figure 3:**
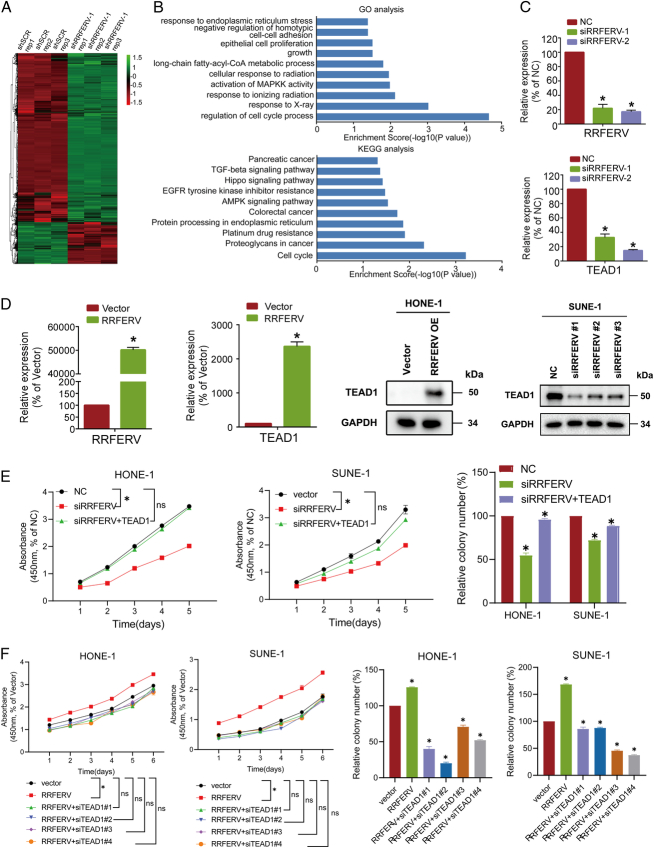
RRFERV stabilizes the transcription of TEAD1. (A, B) GSEA or GEO pathway analysis in RNA-Seq data from cells knocked down for RRFERV. (C) NPC cells were transient transfected with siRNA-targeted RRFERV, and the expression of RRFERV/TEAD1 was detected using qRT-PCR (left panel) and western blotting (right panel). Data are shown as the mean±SEM or SD. *P<0.05, **P<0.01 (*n*=3 independent experiments). (D) In NPC cells, transients were transfected with plasmids containing RRFERV and vector control; after 48 h the cells were harvested and the expression of TEAD1 was detected by qRT-PCR and western blotting. (E) NPC cells knocked down with siRNA-targeted RRFERV and transient transfected with plasmid contents TEAD1, after 48 h colony formation and CCK-8 assays to detect the proliferation. (F) NPC cells with RRFERV overexpression and transient transfected with siRNA-targeted TEAD1 after 48 h colony formation and CCK-8 assays to detect the proliferation.

To determine the underlying molecular mechanism by which RRFERV regulates NPC proliferation and metastasis, we transiently transfected RRFERV shRNA and control shRNA into NPC cells and then performed RNA-seq. Bioinformatic analysis showed significant differences in mRNA expression profiles in cells after knockdown and overexpression of RRFERV. According to KEGG analysis, the DEGs were mostly enriched for the Hippo signaling pathway and other tumor-related pathways (Fig. 3A, B). However, the expression of TEAD1, a core Hippo signaling pathway member, was significantly reduced in the RRFERV knockdown cells. To further investigate the mechanism by which RRFERV regulates TEAD1 and verify the RNA-seq results, we knocked down and overexpressed the RRFERV in NPC cell lines, including SUNE-1 and HONE-1, respectively. Then, the mRNA and protein expression levels of the YAP–TEAD1 signaling pathway members were assessed using qRT-PCR and western blotting. The results showed that the intracellular TEAD1 expression decreased significantly after RRFERV silencing, while TEAD1 expression increased in cells with high RRFERV expression (Fig. 3C, D). Firstly, whether RRFERV regulates downstream targets and signaling pathways through interaction with binding proteins was assessed. qRT-PCR showed that the RRFERV mRNA was enriched after immunoprecipitation of TEAD1 in an RIP assay (Supplementary Fig. S2A, Supplemental Digital Content 2, http://links.lww.com/JS9/D499). Furthermore, we used fluorescent probes to label the lncRNA and observed that RRFERV could rarely co-localize with TEAD1 in cells according to FISH and immunofluorescence assays (Supplementary Fig. 2B, Supplemental Digital Content 2, http://links.lww.com/JS9/D499). These experiments showed that RRFERV may directly bind the TEAD1 protein. It is likely that this direct binding effect is not sufficient to regulate its expression, which is different from its classical regulation acting as a miRNA sponge and needs further experimental verification. To investigate the function of RRFERV in regulating TEAD1 in the malignant progression of NPC, we overexpressed a TEAD1 rescue plasmid in cells that were knocked down for RRFERV. CCK-8 assay and colony formation assay showed that the TEAD1 rescue plasmid could offset the inhibitory effect of RRFERV silencing on NPC cell proliferation according to CCK-8 and colony formation assays (Fig. 3E). Further knockdown of TEAD1 in cells with high RRFERV expression significantly reversed the upregulated proliferation induced by high RRFERV expression (Fig. 3F).

### miR-615-5p and miR-1293 promote NPC progression by directly interacting with TEAD1

**Figure 4:**
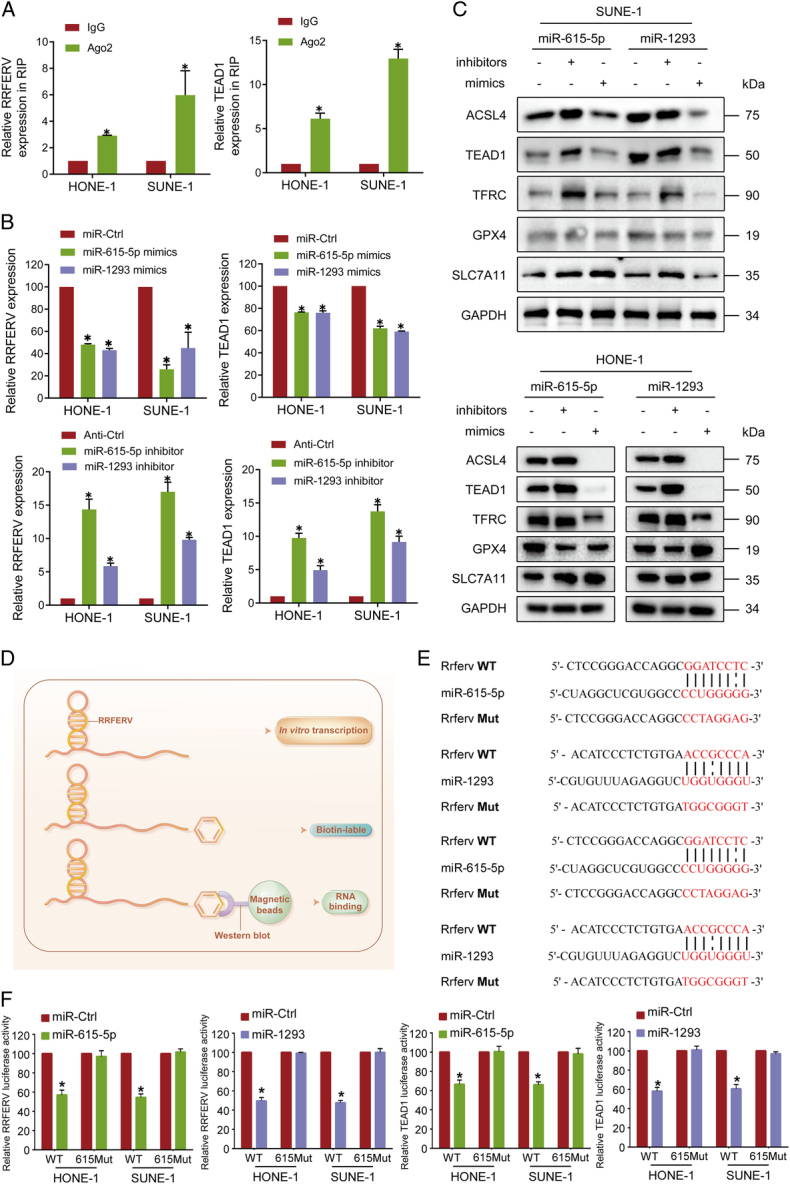
miR-615-5p and miR-1293 promote NPC progression by directly interacting with TEAD1. (A) RRFERV might function as a ceRNA for miRNAs according to an Argonaute 2-based RIP assay. (B, C) NPC cells were transiently transfected with miR-615-5p and miR-1293 mimics and inhibitors; after 48 h cells were harvested, and the expression of the indicated gene was detected by qRT-PCR (B) and western blotting (C). (D) Diagram representing the identification of RRFERV-binding proteins using an *in vitro* transcription followed by an RNA pull-down assay. (E) Dual-luciferase reporter plasmids were constructed by using RRFERV and TEAD1 wild types and mutant sequences as indicated. (F) The RRFERV and TEAD1 dual-luciferase reporter plasmids were constructed and co-transfected two miRNAs with dual-luciferase reporter plasmids in 293T cells, and then the dual-luciferase assay was performed.

LncRNAs can act as ceRNAs to regulate downstream mRNA expression via competitive binding to miRNAs^12,32,33^. To investigate whether RRFERV functions in this way, we performed bioinformatics analysis and found that both RRFERV and TEAD1 are targets for miRNA-615-5p and miR-1293. RIP experiments revealed that RRFERV can directly bind to AgO2, an important protein participating in microRNA-mediated gene silencing (Fig. 4A), suggesting that RRFERV might function as a ceRNA. The Ago2-RIP assay also demonstrated that the RRFERV expression was much higher in miRNA-615-5p or miR-1293 overexpression groups compared with the miR-Ctrl group, indicating that RRFERV and miRNA-615-5p/miR-1293 are in the same RISC. TEAD1 has also been shown to be a miRNA-615-5p/miR-1293 target gene (Supplementary Fig. S3A, Supplemental Digital Content 2, http://links.lww.com/JS9/D499, *P*<0.01). For verification, we overexpressed mimics of miRNA-615-5p and miR-1293 in NPC cells and found that the levels of RRFERV and TEAD1 mRNA were suppressed. Furthermore, we transient transfected inhibitors of the two miRNA into NPC cells, which significantly enhanced the mRNA level RRFERV and the mRNA and protein levels TEAD1 (Fig. 4B, C). Meanwhile, we found that RRFERV knockdown increased the expression of miRNA-615-5p and miR-1293, whereas RRFERV overexpression decreased the levels of miRNA-615-5p and miR-1293 (Supplementary Fig. S3B, Supplemental Digital Content 2, http://links.lww.com/JS9/D499). In addition, we performed RNA pull-down experiments based on *in vitro* transcribed RRFERV followed by western blotting detection of the protein level of TEAD1 (Fig. 4D, Supplementary Fig. S4C, Supplemental Digital Content 2, http://links.lww.com/JS9/D499). The above results tentatively suggested that RRFERV may regulate TEAD1 expression through competitive binding with miR-615-5p/miR-1293 and via direct binding to the mRNA of TEAD1. To further investigate the effect of miR-615-5p and miR-1293 on the direct binding of RRFERV and TEAD1, we constructed RRFERV and TEAD1 dual-luciferase reporter plasmids and co-transfected the two miRNAs with the RRFERV dual-luciferase plasmids in 293T cells (Fig. 4E). Overexpression of the two miRNAs significantly reduced the luciferase signals of RRFERV and TEAD1, indicating that the miRNAs could directly bind to TEAD1 and RRFERV mRNAs to decrease the expression of TEAD1 (Fig. 4F). Moreover, biotin-labeled miRNA pull-down assays increased greatly in RRFERV expression in NPC cells transfected with biotin-labeled miRNA-615-5p and miR-1293 compared with that in the control. Meanwhile, Biotin-labeled miRNA pull-down assays revealed that TEAD1 is the target gene of miRNA-615-5p and miR-1293 (Supplementary Fig. S3D, Supplemental Digital Content 2, http://links.lww.com/JS9/D499, *P*<0.01).

### RRFERV sensitizes NPC to ferroptosis inducers in vitro and in vivo

We showed that exogenous overexpression of RRFERV significantly enhanced NPC cell proliferation and distant metastasis and showed that patients with high RRFERV expression had a poor prognosis. Moreover, RRFERV can upregulate TEAD1 expression through miRNA sponging. Therefore, NPC cells with high RRFERV expression might be tolerant to conventional radiotherapy and chemotherapy. Ferroptosis is characterized by iron overload and lipid peroxidation, and is considered to be an effective anti-cancer strategy^20,23^, especially in tumors that do not respond to conventional chemotherapy and radiotherapy. Epithelial cells that antagonize the E-cadherin-mediated intracellular NRF2–Hippo signaling pathway can promote ferroptosis via YAP^34^, a co-transcriptional activator of TEAD1, which implies that TEAD1 plays an important role in ferroptosis induction. Therefore, we further investigated the effect of RRFERV–TEAD1 on ferroptosis.

**Figure 5:**
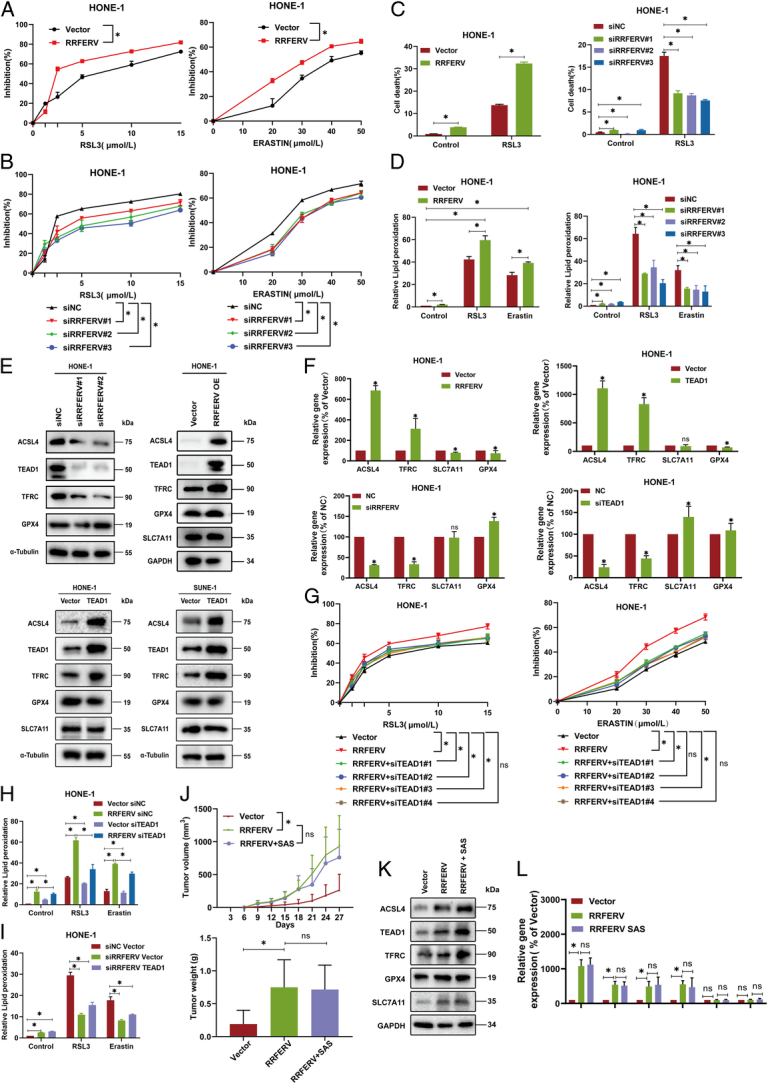
RRFERV sensitizes NPC to ferroptosis inducers *in vitro* and *in vivo*. HONE cells were transiently transfected with plasmid contents RRFERV or vector control; after 24 h, cells were treated with indicated concentration RSL3 or Erastin and the sensitivity of cells was detected by CCK-8. (B) HONE cells were transiently transfected with siRNA-targeted RRFERV or negative control; after 24 h, cells were treated with indicated concentration RSL3 or Erastin and the sensitivity of cells was detected by CCK-8. (C) HONE cells with RRFERV overexpression or knocked down were treated as indicated, and cell viability was detected by flow cytometry. (D) Detection of lipid peroxidation after overexpression or knockdown of RRFERV treated with RSL3/Erastin in HONE cells. (E) Detection of ferroptosis-related gene expression using western blotting after overexpression or knockdown of RRFERV/TEAD1. (F) Detection of the expression levels of ferroptosis-related signal pathway members using qPCR after overexpression or knockdown of RRFERV/TEAD1 in HONE cells. (G) The NPC cells were transient transfected with the indicated plasmids or siRNA; after 24 h the cells were treated with different concentrations of RSLS and Erastin for 24 h, and the CCK-8 assay was performed. (H) After overexpression of RRFERV, TEAD1 was knocked down and lipid peroxidation was detected using BODIPY 581/591 C11 staining. (I) After the knockdown of RRFERV, TEAD1 was overexpressed and lipid peroxidation was detected by using BODIPY 581/591 C11 staining. (J) The SUNE-1 cells stably transfected with the above plasmids were inoculated subcutaneously into BALB/c female nude mice to construct cell-derived xenograft (CDX) in nude mice and injected with sulfasalazine (50 mg/kg/3 days) or not. (K) Detection of ferroptosis-related markers in tumor tissues using western blotting. (L) Detection of ferroptosis-related markers in tumor tissues using qRT-PCR.

We used two different inducers of ferroptosis, RSL3 and Erastin, to treat NPC cells, and found cells with high RRFERV expression were more sensitive to the two inducers (Fig. 5A, Supplementary Fig. S4A, Supplemental Digital Content 2, http://links.lww.com/JS9/D499). By contrast, RRFERV knockdown cells were tolerant to RSL3 and Erastin (Fig. 5B, Supplementary Fig. S4A, Supplemental Digital Content 2, http://links.lww.com/JS9/D499). Similarly, we also observed that cells with high TEAD1 expression were more sensitive to the ferroptosis inducers RSL3 and Erastin, whereas TEAD1 knockdown cells were tolerant to the two inducers (Supplementary Fig. S4B, C, Supplemental Digital Content 2, http://links.lww.com/JS9/D499). Furthermore, overexpression of RRFERV could significantly upregulate the cell death induced by RSL3/Erastin, while its knockdown had the opposite effect by using PI single staining (Fig. 5C). In addition, we also used two kinds of ferroptosis inhibitor with different mechanisms and found that they could effectively reverse the cell death induced by high expression of RRFERV, further confirming that high expression of RRFERV induces cell ferroptosis (Supplementary Fig. S4D, Supplemental Digital Content 2, http://links.lww.com/JS9/D499). Moreover, overexpression of TEAD1 could upregulate RSL3/Erastin-induced cell death, which can be alleviated by TEAD1 knockdown. (Supplementary Fig. S4E, Supplemental Digital Content 2, http://links.lww.com/JS9/D499). Lipid peroxidation characterization by C11BODIPY staining showed that RRFERV overexpression enhanced the lipid peroxidation induced by the ferroptosis inducer, while RRFERV knockdown had the opposite effect (Fig. 5D, Supplementary Fig. S4F, Supplemental Digital Content 2, http://links.lww.com/JS9/D499). And TEAD1 overexpression also enhanced the level of lipid peroxidation induced by ferroptosis inducers and TEAD1 knockdown shows the opposite trend (Supplementary Fig. S4G, Supplemental Digital Content 2, http://links.lww.com/JS9/D499). These results suggested that NPC cells with high RRFERV expression are in higher oxidative stress response and more likely to undergo ferroptosis. To investigate the molecular mechanism of ferroptosis mediated by RRFERV, we detected the expression of key genes in the ferroptosis signaling pathway after RRFERV overexpression in NPC cells. The results showed that the expression levels of ACSL4 and TFRC, which promote ferroptosis, were significantly upregulated at both the mRNA and protein levels (Fig. 5E, F). The YAP–TEAD1 signaling pathway can directly transcribe and activate ACSL4^20^, TFRC, and other genes to activate ferroptosis; therefore, we speculated that the ferroptosis induced by RRFERV overexpression is probably mediated by TEAD1. After TEAD1 overexpression in NPC cells, we detected the expression levels of genes in the ferroptosis-related signaling pathway. We found that ferroptosis-promoting proteins (TFRC and ACSL4) were upregulated in the presence of TEAD1 overexpression (Fig. 5E). In contrast, the expression levels of TFRC and ACSL4 were deregulated after TEAD1 knockdown in NPC cells (Supplementary Fig. S4H, I, Supplemental Digital Content 2, http://links.lww.com/JS9/D499). Cell Counting Kit-8 (CCK-8) and colony formation assays revealed that knockdown of TEAD1 increased the proliferation ability of NPC cells (Fig. 5G, all *P*<0.01, Supplementary Fig. S5J, Supplemental Digital Content 2, http://links.lww.com/JS9/D499). GPX4, SLC7A11, and other ferroptosis suppressor genes were not significantly affected by TEAD1 alterations. To further determine that the activation of the ferroptosis-related signaling pathway mediated by RRFERV acts through YAP–TEAD1 signaling, we used siRNAs to knockdown TEAD1 in NPC cells that overexpressed RRFERV. We found that ACSL4 and TFRC mRNA and protein levels decreased significantly. Furthermore, flow cytometry showed that the level of lipid peroxidation was upregulated by RRFERV overexpression and downregulated after RRFERV knockdown, and all were rescued by TEAD1 alterations (Fig. 5H, I).

RSL3 and Erastin show low solubility in aqueous solution; therefore, we chose SAS as the inducer of ferroptosis *in vivo* ^35^. In the SUNE-1 cell line, we stably overexpressed RRFERV and an empty vector control and then subcutaneously inoculated the tumor cells in the armpits of nude mice. After tumor growth, we administered the drug by gavage 50 mg/kg/2 days. We found that tumors with RRFERV overexpression grew faster. However, the volume and weight of tumors decreased after SAS was added, but the results were not significant (Fig. 5J). In order to analyze the reasons for the poor antitumor effect of SAS, we further collected protein and RNA from tumor tissues. Overexpression of RRFERV effectively induced the expression of TFRC and ACSL4, two ferroptosis-promoting genes, but this effect was not further enhanced by the addition of SAS (Fig. 5K, L). These results suggest that SAS alone may not be effective in inducing ferroptosis in tumor cells. We further detected the co-localization of RRFERV and TEAD1 using FISH and immunofluorescence with the addition of RSL3. Therefore, the above series of experiments proved that NPC with high expression of RRFERV is more likely to undergo ferroptosis via a mechanism related to the transcriptional activation of ACSL4 and TFRC induced by TEAD1.

### RRFERV mediates NPC radiotherapy resistance and renders tumor cells vulnerable to ferroptosis

**Figure 6:**
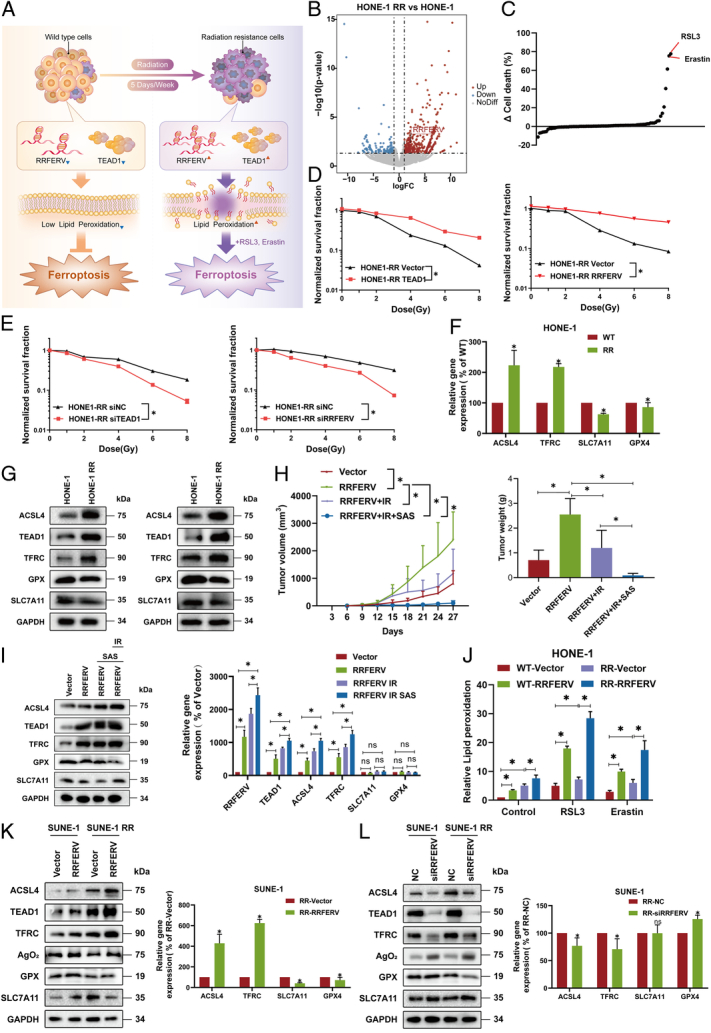
RRFERV mediates NPC radiotherapy resistance and renders tumor cells vulnerable to ferroptosis. (A) A model map of the process of constructing radiotherapy-tolerant cell lines. (B) RNA-seq datasets from paired radiotherapy-tolerant cell lines (HONERR and HONE) were analyzed on 18 pairs of nasopharyngeal carcinoma and normal tissue samples. (C) In the cell death experiment with the HONERR cell line, 80 drugs were screened for inducing cell death and subsequently used in the experiment, followed by flow cytometry analysis to detect cell death. (D) RRFERV and TEAD1 were overexpressed separately, and radiosensitivity was detected by clone formation assays. (E) Radiation resistance NPC cells were transiently transfected siRNA-targeted RRFERV, TEAD1, or negative control; radiosensitivity was detected by clone formation. (F) The expression of TEAD1, RRFERV, and ferroptosis-related genes in radiotherapy-tolerant and wild-type cells was compared by QRT-PCR assay. (G) The expression of TEAD1, RRFERV, and ferroptosis-related genes in radiotherapy-tolerant and wild-type cells was compared by Western blotting assay. (H) SUNE-1 cells with RRFERV overexpression were subcutaneously injected into node mice. When the tumors had grown to a palpable size, the mice were grouped randomly into four groups, as indicated in the figure, and injected intraperitoneally with sulfasalazine (50 mg/kg/3 days), or treated with 6 Gy ionizing radiation, the volume of the tumor was measured every 3 days. After one month, tumors were dissected and weighed. (I) Tumor protein was collected and the western blotting was performed by using the indicated antibodies. Tumor RNA was collected and the QPCR assay was performed by using the indicated primers. (J) NPC wild-type or radiation resistance cells were transiently transfected with plasmids containing RRFERV or vector control; after 24 h, the cells were treated with RSL3 or Erastin. And lipid peroxidation was detected using flow cytometry. (K) The effect of overexpression of RRFERV on the expression of ferroptosis-related factors was detected by using western blotting and qRT-PCR in SUNE and SUNERR cells. (L) The effect of knocking down RRFERV on the expression of ferroptosis-related factors was detected by using western blotting and qRT-PCR in HONE and HONERR cells.

Previously, we found that radiation and DNA damage-related pathways were enriched in NPC patients with knocking down of RRFERV using whole transcriptome sequencing, suggesting that RRFERV might mediate NPC RT resistance (Fig. 3A, B). To investigate the mechanism of RT resistance in NPC in-depth, we simulated the therapeutic regimen commonly used in clinical practice by continuously irradiating the nasopharyngeal carcinoma cell lines SUNE-1 and HONE-1 at a dose of 2 Gy/day until the total radiation dose reached 50 Gy (Fig. 6A). We confirmed the successful cultivation of radiation-tolerant cell lines through colony formation assays (Supplementary Fig. S5A, Supplemental Digital Content 2, http://links.lww.com/JS9/D499). RNA-seq datasets from paired radiotherapy-tolerant (HONERR and HONE) and sensitive (SUNERR and SUNE) cell lines, along with Arraystar LncRNA Microarray 3.0 chip analysis, were conducted on 18 pairs of nasopharyngeal carcinoma and normal tissue samples. We identified a significant difference in the expression of RRFERV, with its expression markedly increased in radiotherapy-tolerant cells. This result suggests that inducing ferroptosis may reverse radiotherapy resistance in nasopharyngeal cancer cells (Fig. 6B, Supplementary Fig. S5B, Supplemental Digital Content 2, http://links.lww.com/JS9/D499). In the cell death experiment with HONERR cells, we screened a collection of 80 drugs specifically designed for inducing cell death. Flow cytometry results revealed that RSL3 and ERASTIN significantly induced cell death in the cell line (Fig. 6C). To verify the role of the RRFERV–TEAD1 signaling axis in the RT tolerance process in NPC, we overexpressed and knocked down RRFERV and TEAD1 in NPC radiotherapy wild-type and tolerant cell lines using transient overexpression and siRNA knockdown methods, respectively. Using radiation treatment with increasing dose gradients, we found that overexpression of RRFERV and TEAD1 significantly reduced the sensitivity of NPC cell lines to RT (Fig. 6D), while knockdown of both genes reversed RT tolerance (Fig. 6E). Furthermore, we compared the expression of RRFERV and TEAD1 in radiotherapy-sensitive and tolerant NPC cells, which showed that the mRNA and protein expression levels of both genes were markedly upregulated in RT-tolerant NPC cells compared with those in the sensitive cell lines (Fig. 6F, G, Supplementary Fig. S5C, D, Supplemental Digital Content 2, http://links.lww.com/JS9/D499). These results suggested that the activation of RRFERV–TEAD1 is the key to mediating radiotherapy in NPC.

Ferroptosis-mediated programmed cell death relies on lipid peroxidation but not caspases, which is very different from apoptosis. We found that the expression levels of TFRC and ACSL4, the key genes that mediate ferroptosis, were upregulated in RT-tolerant cell lines compared with those in sensitive cell lines (Supplementary Fig. S5E, Supplemental Digital Content 2, http://links.lww.com/JS9/D499). We used two ferroptosis inducers, RSL3 and Erastin, to treat RT-resistant NPC cells. The ferroptosis inducers significantly induced lipid peroxidation in radiotherapy-resistance cells (Supplementary Fig. S5F, Supplemental Digital Content 2, http://links.lww.com/JS9/D499). Therefore, *in vivo* animal experiments result (Fig. 5J) showed that SAS alone had no significant effect on the growth of NPC transplanted tumors, while the tumors in mice regressed significantly after the combined application of RT and SAS, which was more effective compared with RT alone (Fig. 6H). We further collected the protein and RNA from the tumor tissues for Western and QPCR. The results showed that SAS combined with radiotherapy effectively induced high expression of ferroptosis-promoting genes TFRC and ACSL4, which further induced ferroptosis in tumor tissues overexpressing RRFERV (Fig. 6I). Then, we wanted to know how RRFERV–TEAD1 signaling regulates the vulnerable state of lipid peroxidation in RT-resistant NPC cells. We knocked down and overexpressed RRFERV in radiotherapy-resistant NPC cells, respectively, and found that the lipid peroxidation level and ferroptosis sensitivity of the cells increased after RRFERV overexpression and decreased after RRFERV knockdown (Fig. 6J, Supplementary Fig. S5G, H, Supplemental Digital Content 2, http://links.lww.com/JS9/D499). Moreover, TEAD1 is a similar phenomenon (Supplementary Fig. S5I, J, Supplemental Digital Content 2, http://links.lww.com/JS9/D499). This indicated that the RT-tolerant NPC cell lines were in a fragile oxidative stress state and might be more prone to ferroptosis induction. Furthermore, in RT-tolerant NPC cells, western blotting and qRT-PCR showed that RRFERV overexpression significantly upregulated the levels of ferroptosis-promoting factors TFRC and ACSL4, while ferroptosis inhibiting proteins SLC7A11 and GPX4 showed a certain decrease (Fig. 6K, Supplementary Fig. 5K, Supplemental Digital Content 2, http://links.lww.com/JS9/D499). In addition, knocking down RRFERV significantly reduced the expression of ferroptosis-promoting factors (Fig. 6L, Supplementary Fig. S5L, Supplemental Digital Content 2, http://links.lww.com/JS9/D499). The above results indicated that the RRFERV–TEAD1 signal axis might promote cell lipid peroxidation by directly transcribing and activating ferroptosis-promoting genes such as TFRC and ACSL4 to mediate NPC radiotherapy tolerance, resulting in NPC radiotherapy-resistance cells being more prone to ferroptosis. Therefore, we propose that ferroptosis inducers could be applied in patients with NPC with RT resistance, breaking the balance of tumor lipid peroxidation and reversing radiation resistance.

## Discussion

**Figure 7:**
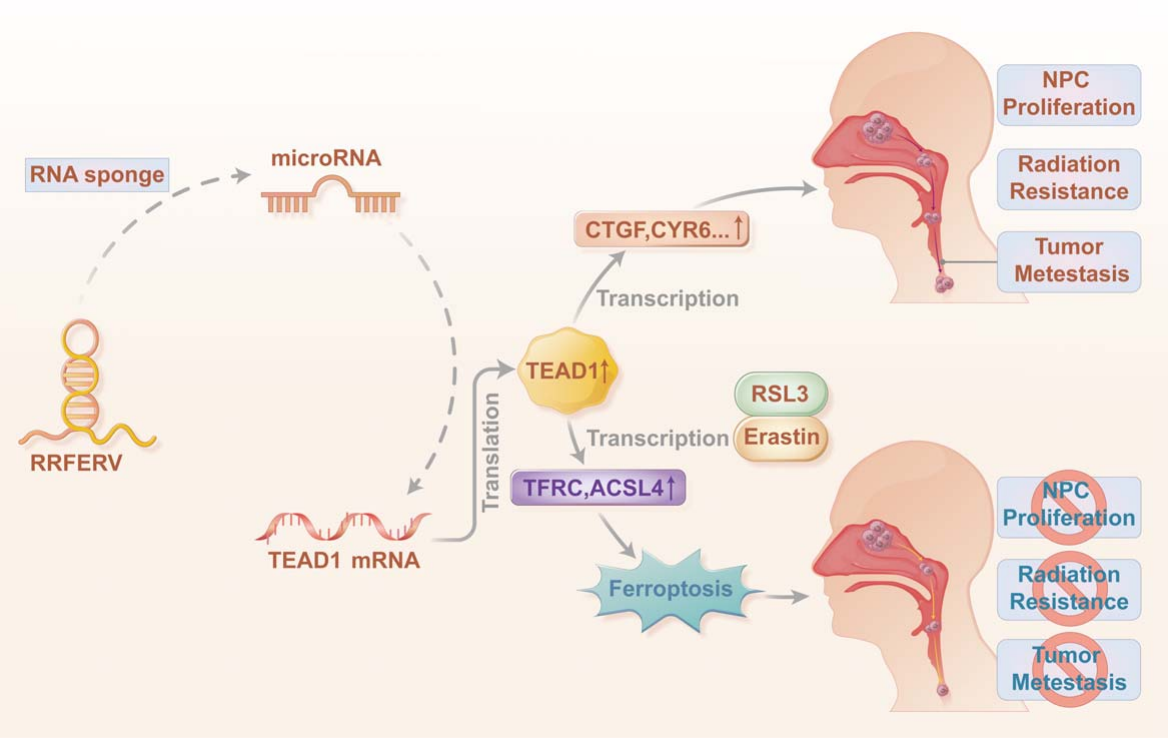
Diagram showing the mechanism of RRFERV promoting NPC proliferation, metastasis, and radiation resistance.

In this study, we found that the lncRNA RRFERV was highly expressed in NPC tissues but not in normal tissues, and its expression correlated negatively with NPC prognosis. We revealed that RRFERV-mediated TEAD1 upregulation promoted significant NPC cell proliferation, invasion, and metastasis. Our study further revealed that RRFERV could compete with miRNA-615-5p and miR-1293 to bind TEAD1 mRNA, acting as a miRNA sponge, thereby regulating the expression of TEAD1. In addition, RRFERV could mediate RT resistance in NPC. At the same time, oxidative stress was in a fragile balance in RT-resistant cells, making them more susceptible to ferroptosis inducers. Mechanistically, the RRFERV–TEAD1 axis mediates the transcription of ACSL4 and TFRC. Additionally, we identified a feedback inhibitory pathway such that NPC cells with high RRFERV expression, on the one hand, induce the TEAD1 pathway and promote the malignant progression of NPC; on the other hand, RRFERV makes NPC cells more prone to ferroptosis, which might be related to the activation of genes such ACSL4 and TFRC by TEAD1. These results indicate a new direction for the treatment of patients with NPC, especially for those who are resistant to conventional treatment (Fig. 7).

With a high proportion in the total transcriptome and similar structures and sequence compositions to mRNA, it is clear that lncRNAs are not the ‘silent majority’^36,37^. LncRNAs have various functions in processes such as genome scaffold formation, transcriptional control, regulation of transcript stability, and translation control^38–42^, which broadly influence the physiological and biochemical activities of cells, especially in tumors^43,44^. It has been reported in the literature that lncRNAs can act as competing endogenous RNAs (ceRNAs) to sponge microRNAs (miRNAs) during transcriptional regulatory processes^12^. miRNA plays an important role in the post-transcriptional regulation of mRNA. The mechanism is the miRISC (miRNA-induced silencing complex) induces the decay of mRNA and translational suppression through the interaction with the complementary sequences in the 3′-untranslated region (3′-UTR) of target gene mRNA^45^. Therefore, the dysregulation of miRNA expression is closely associated with cancer initiation, progression, and metastasis. However, recent studies report that certain lncRNAs exert their biological functions by interacting with proteins^46,47^. Although RIP assays revealed that RRFERV can bind with TEAD1, FISH showed that TEAD1 (red) localizes to the nucleus and RRFERV (green) to the cytoplasm, and there is less co-localization between the two in cells. It is likely that this direct effect is not sufficient to regulate its expression, and ceRNA is the key mechanism of regulation. Herein, we revealed that RRFERV, acting as a miRNA sponge, can compete with miRNA-615-5p and miR-1293 to bind TEAD1 mRNA, thereby regulating the expression of TEAD1.

The TEAD1 signaling pathway has a critical role in growth and development, and is responsible for the contact inhibition that occurs in normal tissues, thereby controlling histogenesis. In contrast, excessive activation of TEAD1 in tumor cells can deprive cells of contact inhibition, resulting in proliferation and apoptosis resistance of tumor cells^34^. TEAD1 promotion of anti-apoptotic gene transcription means that YAP–TEAD1-activated tumor cells are difficult to kill via apoptosis-inducing strategies such as radiotherapy and/or chemotherapy. TEAD1 can also induce the cell cycle process, which considerably accelerates tumor progression^48–50^. Ferroptosis is a recently discovered pathway for non-apoptotic cell death, which occurs via a mechanism of sustained peroxidation of polyunsaturated fatty acids, which causes irreversible damage to the structure of the cellular phospholipid membrane system that eventually ruptures the cell^51–53^. We found that NPC cells with high RRFERV expression were more susceptible to the ferroptosis inducers RSL3 and Erastin, whereas, after RRFERV knockdown, the cells became resistant to them. Moreover, the addition of sulfasalazine, another inducer of ferroptosis, significantly inhibited the proliferation of tumors in the group with high RRFERV expression in an *in vitro* subcutaneous tumor model in nude mice. A lot of experiments have proved that the level of lipid peroxidation was upregulated by RRFERV overexpression and downregulated after RRFERV knockdown, and all were rescued by TEAD1 alterations. Mechanically, we found that the expression levels of two key genes promoting ferroptosis, TFRC and ACSL4, were significantly upregulated upon TEAD1 overexpression and downregulated upon TEAD1 knockdown, while we detected the same trend in their mRNA expression. Therefore, we concluded that TEAD1, as a downstream molecule of RRFERV, not only upregulates lipid peroxidation levels in cells but also transcriptionally activates ferroptosis-promoting factors TFRC and ACSL4, which render cells vulnerable to ferroptosis.

The clinical treatment of NPC mainly relies on radiotherapy; however, about one-third of patients cannot benefit from it, in which the main cause of treatment failure is radiotherapy-resistance. Previous studies on radiotherapy resistance mainly focused on DNA damage caused by radiotherapy, as well as the associated caspase activation and apoptosis. Lei *et al*.^35^ found that radiotherapy could induce ferroptosis by mediating the transcription of SLC7A11 to cause the accumulation of lipid peroxidation, indicating that ferroptosis has an important function in the RT process; however, the association between RT resistance and ferroptosis has not been reported. To further determine the molecular mechanism of RT resistance in NPC, we constructed a cellular model of RT resistance and found that the radioresistant cells had a significantly higher lipid peroxidation level than the wild-type cells, suggesting that radiotherapy-resistant cells were in a fragile oxidative stress equilibrium. There have been increasing reports on the regulatory relationship between lncRNA and ferroptosis in cancers in recent years. LncRNA p53RRA interacts with G3BP1 in order to promote ferroptosis in lung cancer through nuclear isolation of p53^54^. NEAT1 modulates the miR-362-3p–MIOX axis in order to promote ferroptosis in HCC cells^55^. LINC00336 inhibits ferroptosis by regulating the expression of cystathionine-β-synthase and serving as an endogenous sponge of miR-6852 in lung cancer^56^. Moreover, lncRNA OIP5-AS1 targets the miR-128-3p–SLC7A11 pathway to inhibit ferroptosis in prostate cancer^57^. Our study revealed a critical regulatory role of RRFERV in the malignant progression of NPC. RT combined with a ferroptosis inducer has good application prospects in the treatment of patients with recurrent and refractory NPC.

## Conclusions

Our findings suggest that RRFERV serves as an independent prognostic factor in NPC. During the malignant progression of NPC caused by high expression of RRFERV, ferroptosis can be induced to effectively kill cancer cells and reverse the radiotherapy resistance of NPC cells, suggesting that recurrent and refractory NPC might be treated by inducing ferroptosis.

## Ethical approval

This study received approval from the Institutional Review Board of Sun Yat-Sen University Cancer Center (SYSUCC). The conjunctive number is B2022-599-01.

## Consent

All patients provided written informed consent.

## Source of funding

This research was funded by the Innovative Research Group Project of the National Natural Science Foundation of China (82003214, 81802789, 82373067), Natural Science Foundation of Guangdong Province (2021A1515010853), Guangzhou Municipal Science and Technology Program key projects (2023A04J1771), Guangdong Basic and Applied Basic Research Foundation (2019A1515110076) and Funding by Science and Technology Projects in Guangzhou (2024A04J5234), the Fundamental Research Funds for the Central Universities, Sun Yat-sen University, Clinical Research 5010 Program (202208).

## Author contribution

L.C., Q.X., and X.W.: conceived the experiments; C.H., Z.L., Y.C., L.L., Z.H., J.L., and C.S.: carried out and analyzed the data for most of the *in vitro* experiments; S.S., Y.C., L.C., Z.X., and J.L.: collected NPC samples and performed the IHC experiments; Q.X., X.L., L.L., and L.C.: designed and performed the animal experiments; Z.H., C.H., C.S., S.Z., Z.L., and Z.X.: helped with the data analyses; Q.X., Y.C., L.C., S.S., and X.L.: wrote and revised the manuscript; X.L., L.C., Q.X., S.Z., and X.W.: supervised the study. All authors reviewed and discussed the final version of the paper.

## Conflicts of interest disclosure

The authors declare no conflicts of interest.

## Research registration unique identifying number (UIN)

1. Name of the registry: Research Registry.
2. Unique identifying number or registration ID: researchregistry10040.

## Guarantor

Lei Chen.

## Data availability statement

The data that support the findings of this study are available from the corresponding author upon reasonable request.

## Provenance and peer review

Not applicable.

## Supplementary Material
