# Supplementary material for: RRFERV stabilizes TEAD1 expression to mediate nasopharyngeal cancer radiation resistance rendering tumor cells vulnerable to ferroptosis
Source: Int J Surg. 2024 Sep 30;111(1):450–66. doi: 10.1097/JS9.0000000000002099 (PMC11745583; doi:10.1097/JS9.0000000000002099)
Supplement: Supplementary file 1 [file js9-111-0450-s001.docx]

**Tables**

**Table 1.** List of primers used in this study.

**Gene RT-PCR**

# shRNA target sequences

RRFERV-homo-26 RRFERV-homo-256 RRFERV-homo-383 RRFERV-homo-520 TEAD1-homo-950 TEAD1-homo-1018 TEAD1-homo-685 TEAD1-homo-1826

# microRNA mimics and inhibitors

hsa-miR-615-5p mimics hsa-miR-615-5p inhibitor hsa-miR-1293 mimics hsa-miR-1293 inhibitor **qPCR sequences**

Tead1 Q1 F

Tead1 Q1 R Tead1 Q2 F Tead1 Q2 R GAPDH QPCR F GAPDH QPCR R RRFERV RRFERV RRFERV RRFERV

ACSL4-F ACSL4-R TFRC-F TFRC-R GPX4-F GPX4-R SLC7A11 SLC7A11 SLC7A11 SLC7A11

**Sequence(5’ to 3’)**

GCAGGAACAAGGGAGAGCUTT CCGUGGAGAUGACGCUGAATT UCCUACAUCCCUCUGUGAATT GCCUGGGAAGCUGGAAACATT GGAUCAGACUGCAAAGGAUTT CCACUGCCAUUCAUAACAATT CUGCAGAUAAGCCAAUUGATT CAUGGCCUGUGUGUUUGAATT

GGGGGUCCCCGGUGCUCGGAUC GAUCCGAGCACCGGGGACCCCC UGGGUGGUCUGGAGAUUUGUGC GCACAAAUCUCCAGACCACCCA

AACTCAGGACAGGCAAGACG GGCTTGACGTCTTGTGAGGA GGAAAGGGCCCTCAAAATGC ACACATTGGGGAGCGGTTTA CGCTGAGTACGTCGTGGAGTC GCTGATGATCTTGAGGCTGTTGTC AAGCTGTACCACTTCGAGGG ACCAGCTCACAGAGGAACAC ACGTTCCTACATCCCTCTGTG GGTGTTTCCAGCTTCCCAGG GCTATCTCCTCAGACACACCGA AGGTGCTCCAACTCTGCCAGTA ATCGGTTGGTGCCACTGAATGG ACAACAGTGGGCTGGCAGAAAC ACAAGAACGGCTGCGTGGTGAA GCCACACACTTGTGGAGCTAGA TGGTCAGAAAGCCTGTTGTGT TGCTCCAATGATGGTGCCAA TCATGGTTGCCCTTTCCCTC TCTTCTTCTGGTACAACTTCCAGT

**Table 2.** Clinicopathological characteristics of studied patients and expression of RRFERV in 220 patients

with nasopharyngeal carcinoma

| **Characteristic** | **No. of patients** | **(%)** |
| --- | --- | --- |
| **Age** |  |  |
| ≤ 45 years | 104 | 47.3 |
| > 45 years | 116 | 52.7 |
| **Gender** |  |  |
| Female | 54 | 24.5 |
| Male  **WHO Type** | 166 | 75.5 |
| II | 0 | 0 |
| III | 220 | 100 |
| **VCA-IgA** |  |  |
| < 1:80 | 23 | 10.5 |
| ≥ 1:80 | 197 | 89.5 |
| **EA-IgA** |  |  |
| < 1:10 | 38 | 17.3 |
| ≥ 1:10 | 182 | 82.7 |
| **T Stage** |  |  |
| T1-T2 | 39 | 17.7 |
| T3-T4 | 181 | 82.3 |
| **N Stage** |  |  |
| N0-N1 | 110 | 50.0 |
| N2-N3  **TNM Stage** | 110 | 50.0 |
| III | 137 | 62.3 |
| IV | 83 | 37.7 |

**Locoregional failure**

No 180 81.8

Yes 40 18.2

**Distant metastasis**

| No | 161 | 73.2 |
| --- | --- | --- |
| Yes | 59 | 26.8 |
| **Death** |  |  |
| No | 138 | 62.7 |
| Yes | 82 | 37.3 |
| **Expression of RRFERV** | | |
| Low expression | 67 | 30.5 |
| High expression | 153 | 69.5 |

Abbreviations: VCA-IgA, viral capsid antigen immunoglobulin A; EA-IgA, early antigen immunoglobulin A. All patients were restaged according to the 8^th^ edition of the AJCC Cancer Staging Manual.

**Table 3.** Correlation between the clinicopathological features and RRFERV expression in 220 patients with nasopharyngeal carcinoma

| **Characteristic** | **No. of patients** | **RRFERV expression** | | ***P* Value*** |
| --- | --- | --- | --- | --- |
|  |  | **Low, *n* (%)** | **High, *n* (%)** |  |
| **Age** |  |  |  |  |
| ≤ 45 years | 104 | 29 (43.3) | 75 (49.0) | 0.433 |
| >45 years | 116 | 38 (56.7) | 78 (51.0) |  |
| **Gender** |  |  |  |  |
| Female | 54 | 16 (23.9) | 38 (24.8) | 0.879 |
| Male | 166 | 51 (76.1) | 115 (75.2) |  |
| **WHO Type** |  |  |  |  |
| II | 0 | 0(0) | 0(0) |  |
| Ⅲ | 220 | 67(100) | 153 (100) |  |
| **VCA-IgA** |  |  |  |  |
| < 1:80 | 23 | 9 (13.4) | 14 (9.2) | 0.339 |
| ≥ 1:80 | 197 | 58 (86.6) | 139 (90.8) |  |
| **EA-IgA** |  |  |  |  |
| < 1:10 | 38 | 12 (17.9) | 26 (17.0) | 0.868 |
| ≥ 1:10 | 182 | 55 (82.1) | 127 (83.0) |  |
| **T Stage** |  |  |  |  |
| T1-T2 | 39 | 8 (11.9) | 31 (20.3) | 0.137 |
| T3-T4 | 181 | 59 (88.1) | 122 (79.7) |  |
| **N Stage** |  |  |  |  |
| N0-N1 | 110 | 39 (58.2) | 71 (46.4) | 0.133 |
| N2-N3 | 110 | 28 (41.8) | 82 (53.6) |  |
| **TNM Stage** |  |  |  |  |
| III | 137 | 46 (68.7) | 91 (59.5) | 0.107 |
| IV | 83 | 21 (31.3) | 62 (40.5) |  |
| **Locoregional failure** | | | | |
| No | 180 | 57 (85.1) | 123 (80.4) | 0.407 |
| Yes | 40 | 10 (14.9) | 30 (19.6) |  |
| **Distant metastasis** |  |  |  |  |
| No | 161 | 57 (85.1) | 104 (68.0) | **0.008** |
| Yes | 59 | 10 (14.9) | 49 (32.0) |  |
| **Death** |  |  |  |  |
| No | 138 | 49 (73.1) | 89 (58.2) | **0.035** |
| Yes | 82 | 18 (19.4) | 64 (41.8) |  |

Abbreviations: VCA-IgA, viral capsid antigen immunoglobulin A; EA-IgA, early antigen immunoglobulin A. Bold values indicate *P*< 0.05, *P* value is determined by χ2. All patients were restaged according to the 8th edition of the AJCC Cancer Staging Manual.

**Table 4.** Univariate and multivariable Cox regression analysis of prognostic factors in 220 patients with nasopharyngeal carcinoma

**Variable Univariate analysis Multivariate analysis**

|  |  |  |  |  |  |  |  |
| --- | --- | --- | --- | --- | --- | --- | --- |
|  | **HR** | **95%CI** | ***P-*valu**  **e** |  | **HR** | **95%CI** | ***P-*value** |
| **Overall survival** |  |  |  |  |  |  |  |
| RRFERV expression (high vs. low) | 1.77 | 1.05-2.99 | **0.032** |  | 1.74 | 1.03-2.94 | **0.040** |
| T stage (T3-T4 vs. T1-T2) | 0.98 | 0.56-1.71 | 0.979 |  |  |  |  |
| N stage (N2-N3 vs. N0-N1) | 2.01 | 1.28-3.15 | **0.002** |  | 2.00 | 1.27-3.15 | **0.003** |
| Age (≥ 45 vs. <45 years) | 1.64 | 1.05-2.57 | **0.031** |  |  |  |  |
| Gender (Male vs. female) | 0.51 | 0.28-0.92 | **0.025** |  |  |  |  |
| VCA IgA (≥ 1:80 vs. < 1:80) | 1.03 | 0.51-2.06 | 0.938 |  |  |  |  |
| EA IgA (≥ 1:10 vs. < 1:10) | 1.10 | 0.62-1.96 | 0.738 |  |  |  |  |
| **Disease-free survival** |  |  |  |  |  |  |  |
| RRFERV expression (high vs. low) | 1.88 | 1.12-3.17 | **0.017** |  | 1.76 | 1.04-2.96 | **0.034** |
| T stage (T3-T4 vs. T1-T2) | 0.73 | 0.44-1.23 | 0.239 |  |  |  |  |
| N stage (N2-N3 vs. N0-N1) | 1.94 | 1.25-2.99 | **0.003** |  | 1.93 | 1.24-2.99 | **0.003** |
| Age (≥ 45 vs. <45 years) | 1.20 | 0.78-1.84 | 0.406 |  |  |  |  |
| Gender (Male vs. female) | 0.67 | 0.39-1.13 | 0.134 |  |  |  |  |
| VCA IgA (≥ 1:80 vs. < 1:80) | 1.00 | 0.50-2.00 | 0.998 |  |  |  |  |
| EA IgA (≥ 1:10 vs. < 1:10) | 1.15 | 0.65-2.04 | 0.627 |  |  |  |  |
| **Distant metastasis-free survival** |  |  |  |  |  |  |  |
| RRFERV expression (high vs. low) | 2.38 | 1.20-4.69 | **0.013** |  | 2.16 | 1.09-4.27 | **0.027** |
| T stage (T3-T4 vs. T1-T2) | 0.65 | 0.36-1.19 | 0.161 |  |  |  |  |
| N stage (N2-N3 vs. N0-N1) | 2.46 | 1.42-4.25 | **0.001** |  | 2.39 | 1.38-4.15 | **0.002** |
| Age (≥ 45 vs. <45 years) | 1.23 | 0.73-2.06 | 0.440 |  |  |  |  |
| Gender (Male vs. female) | 0.65 | 0.34-1.25 | 0.198 |  |  |  |  |
| VCA IgA (≥ 1:80 vs. < 1:80) | 1.07 | 0.46-2.49 | 0.878 |  |  |  |  |
| EA IgA (≥ 1:10 vs. < 1:10) | 1.47 | 0.70-3.09 | 0.314 |  |  |  |  |

Abbreviations: VCA-IgA, viral capsid antigen immunoglobulin A; EA-IgA, early antigen immunoglobulin A. Bold values indicate *P*< 0.05, *P* value is determined by Cox regression analysis. All patients were restaged according to the 8th edition of the AJCC Cancer Staging Manual.
