# Supplementary material for: RRFERV stabilizes TEAD1 expression to mediate nasopharyngeal cancer radiation resistance rendering tumor cells vulnerable to ferroptosis
Source: Int J Surg. 2024 Sep 30;111(1):450–66. doi: 10.1097/JS9.0000000000002099 (PMC11745583; doi:10.1097/JS9.0000000000002099)
Supplement: Supplementary file 2 [file js9-111-0450-s002.docx]

**
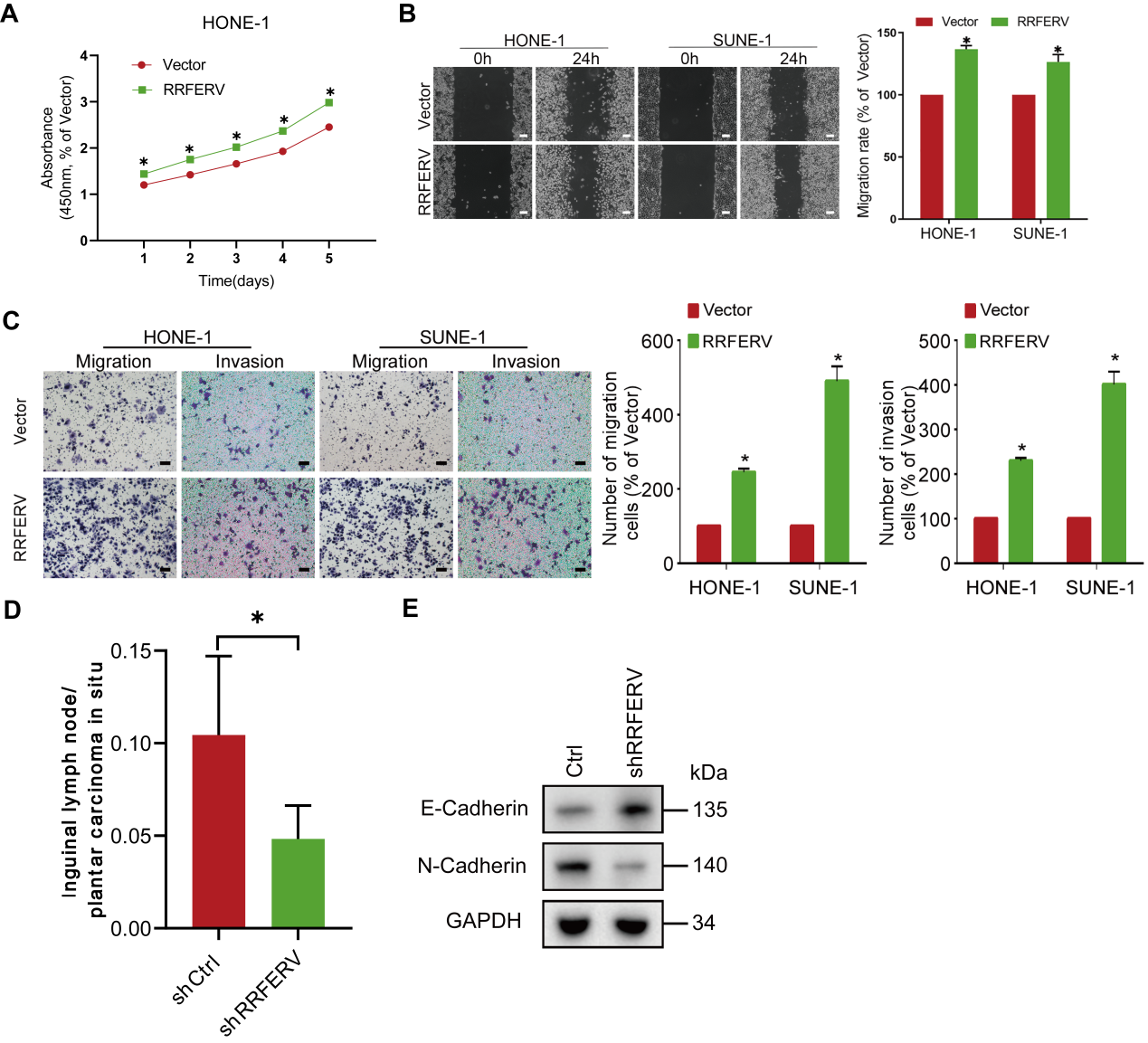
**

**Supplement Figure 2. legends**

**(A-C)** RRFERV overexpression and vector control plasmids were transfected in HONE-1 and SUNE-1 cells, followed by CCK8 assays **(A)**, wound healing assays **(B)**, and transwell assays **(C). (D)** The ratio of inguinal lymph node volume/footpad tumor volume of each nude mouse in the RRFERV knockdown group compared to the ctrl group. **(E)** Western blotting detected the levels of proteins associated with the EMT process.


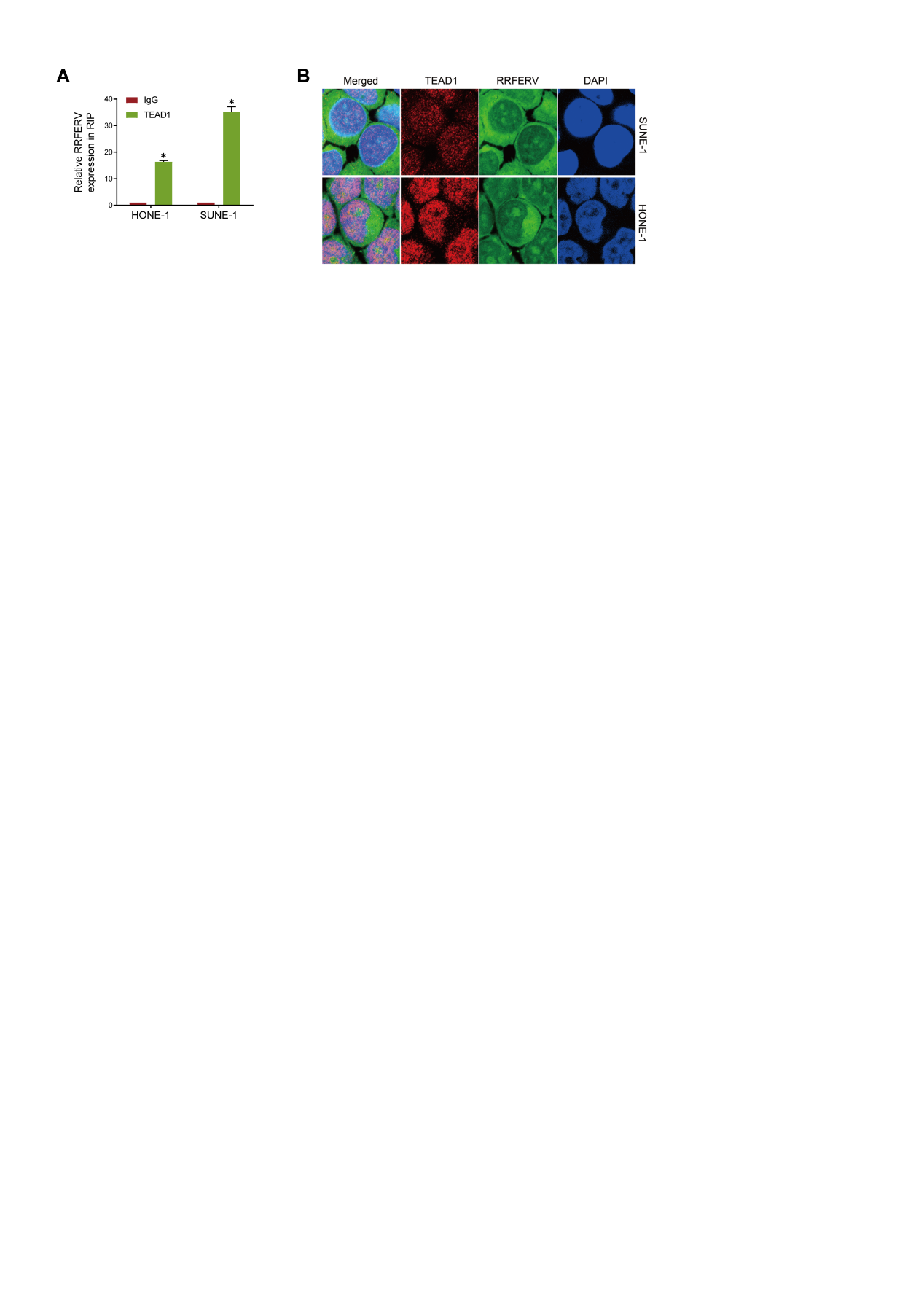


**Supplement Figure 3. legends**

**(A)** A RIP assay was performed to detect the enrichment of RRFERV mRNA after TEAD1 IP, assessed using qRT‑PCR. **(B)** RRFERV could not co-localize with TEAD1 in HONE-1 and SUNE-1 cells in normal condition according to the FISH and immunofluorescence assay.


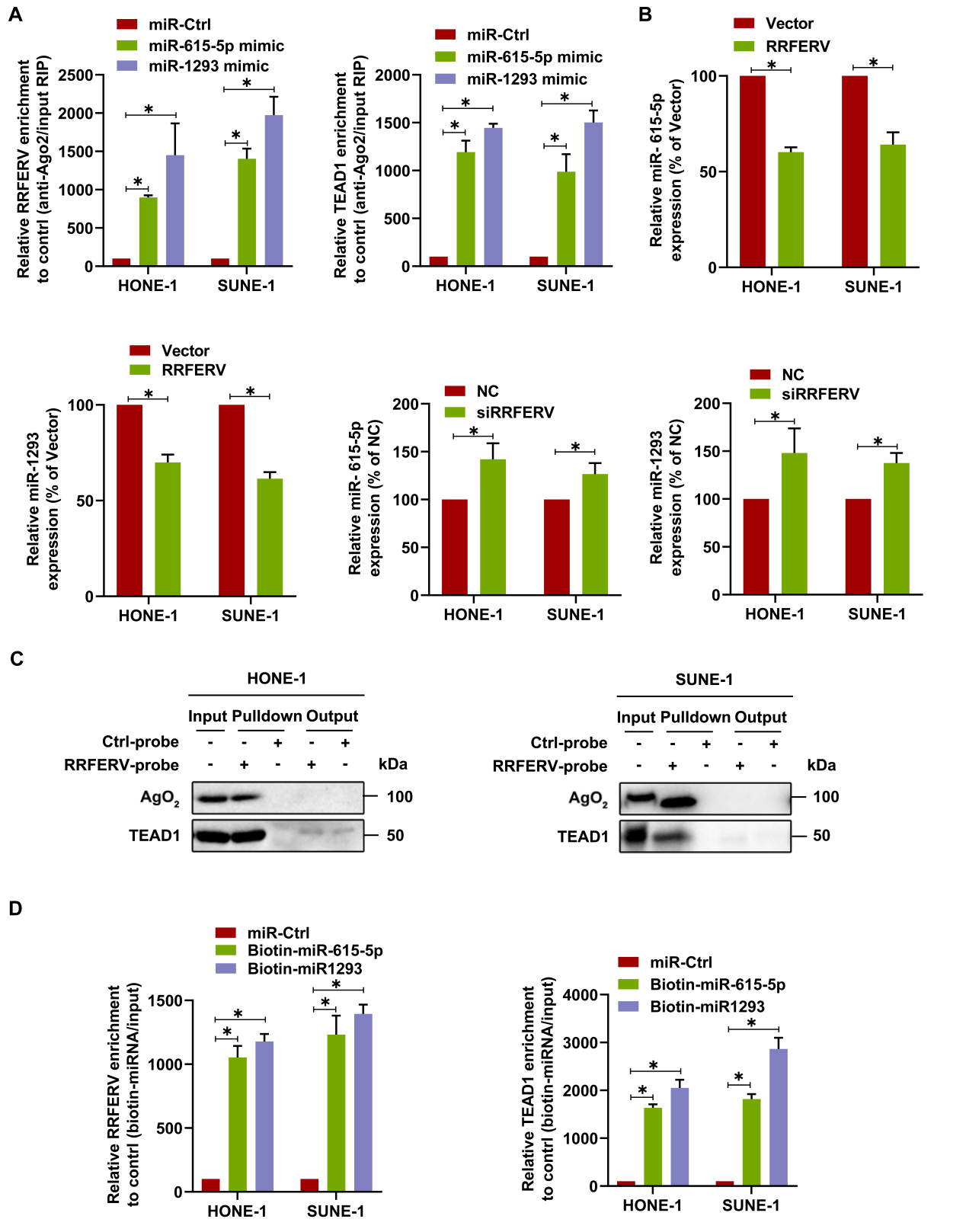


**Supplement Figure 4. legends**

**(A)** Ago2 protein immunoprecipitated by Ago2 antibody or IgG was detected by Western blot analysis transfected with miRNA-615-5p and miR-1293, or miR-Ctrl in HONE-1 and SUNE-1 cells. **(B)** Relatve levels of miRNA-615-5p and miR-1293 in HNE-1 and SUNE-1 cells transfected with sh-RRFERV or RRFERV OE. **(C)** Western blotting detected the protein level when NPC cells were transcribed RRFERV. **(D)** Enrichment of RRFERV or TEAD1 pulled down by miRNA-615-5p and miR-1293, or negative control.

**
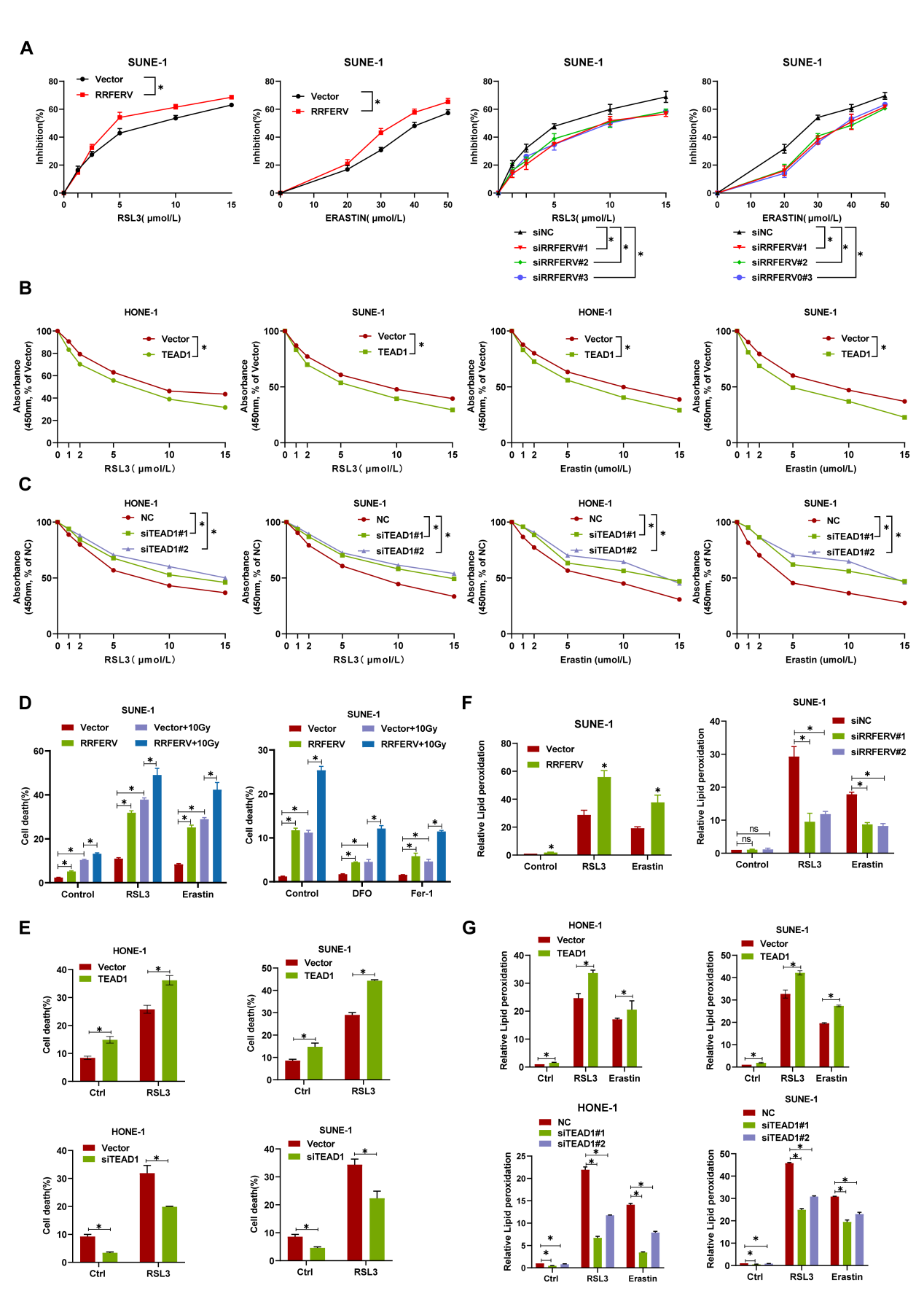
**

**
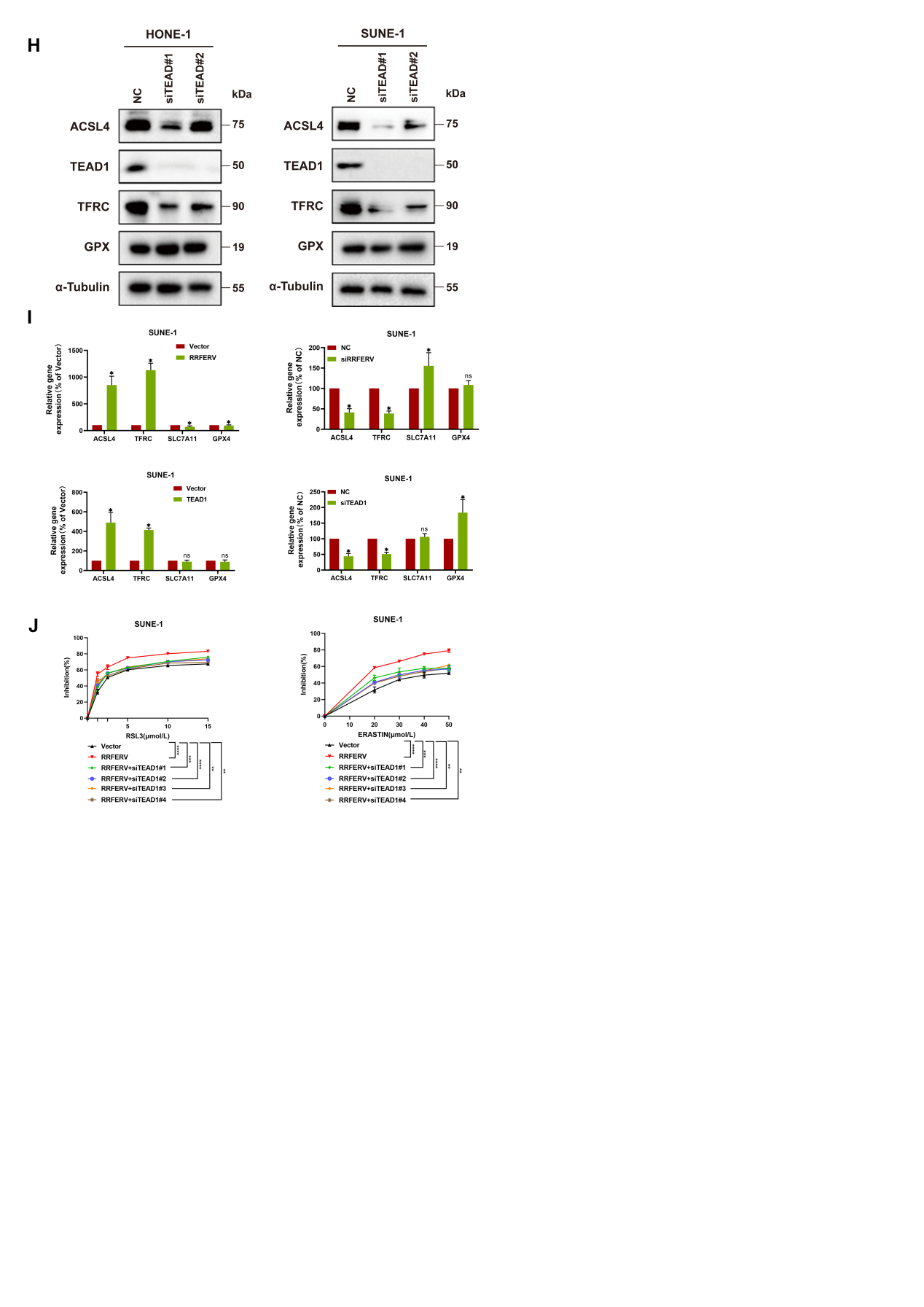
**

**Supplement Figure 5. legends**

**(A)** SUNE cells were transiently transfected with RRFERV overexpression and decreased after RRFERV knockdowncontents, after 24 hours, cells were treated with indicated concentration RSL3 or Erastin and the sensitivity of cells was detected by CCK8. **(B-C)** HONE and SUNE cells were transiently transfected with TEAD1 overexpression and decreased after TEAD1 knockdown, after 24 hours, cells were treated with indicated concentration RSL3 or Erastin and the sensitivity of cells was detected by CCK8. **(D)** SUNE cells were transiently transfected with plasmids contents RRFERV or vector control, after 24 hours, cells were treated with indicated concentration RSL3, Erastin, DFO or Fer-1. Flow cytometer was used to detect cell death with or without IR. **(E)** Flow cytometer was used to detect cell death upon TEAD1 overexpression or knockdown. **(F)** Lipid peroxidation was used to detect cell death upon RRFERV overexpression or knockdown. **(G)** NPC cells were transiently transfected with siRNA targeted TEAD1 or negative control, after 24 hours, cells were treated with indicated concentration RSL3 or Erastin and cell death was detected by lipid peroxidation. **(H-I)** Detection of the expression levels of ferroptosis-related signal pathway members using western blot and qPCR after overexpression or knockdown of RRFERV/TEAD1. **(J)** Counting Kit-8 (CCK-8) revealed that knockdown of TEAD1 increased the proliferation ability of SUNE cells with indicated concentration RSL3 or Erastin.

**
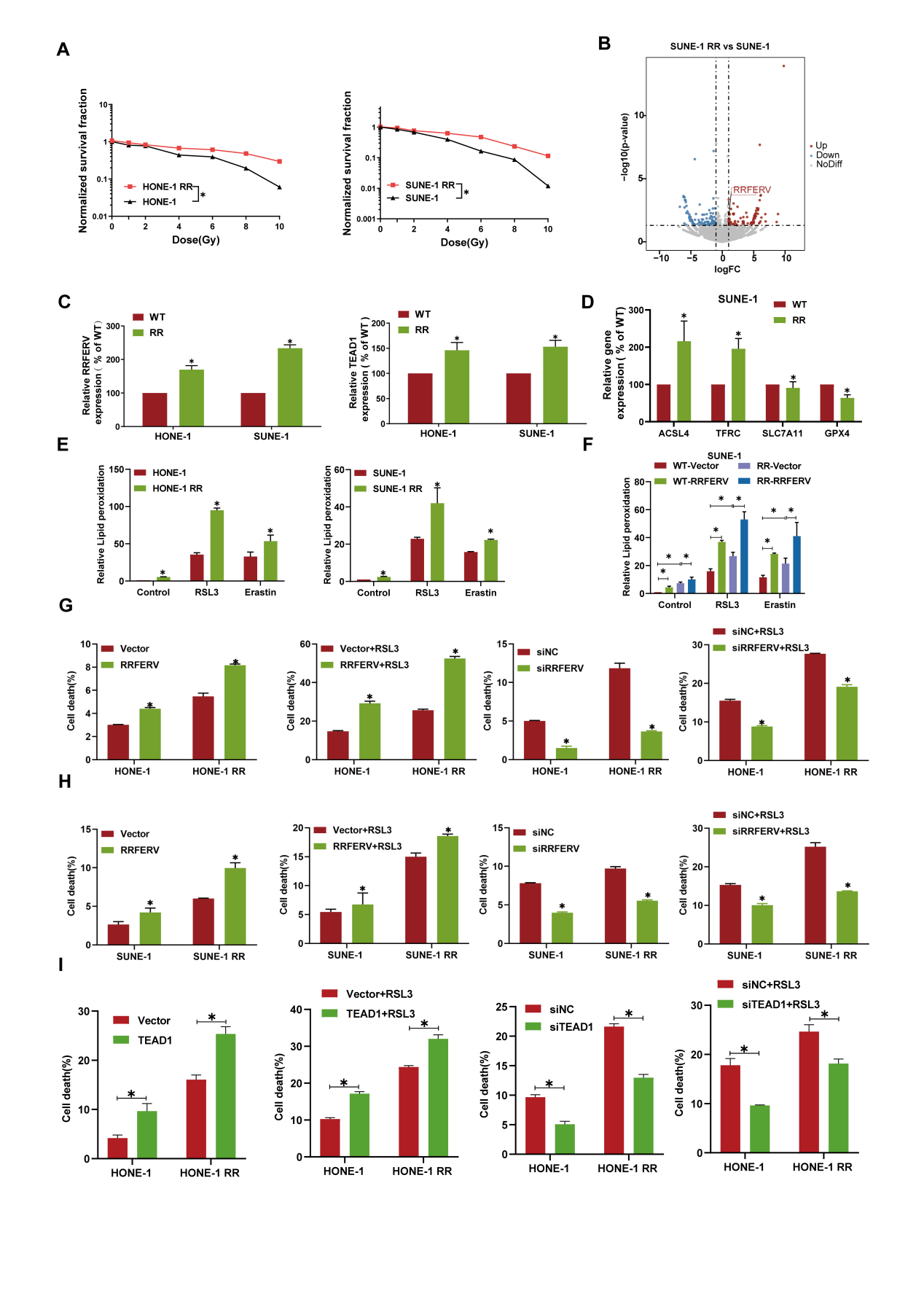
**

**
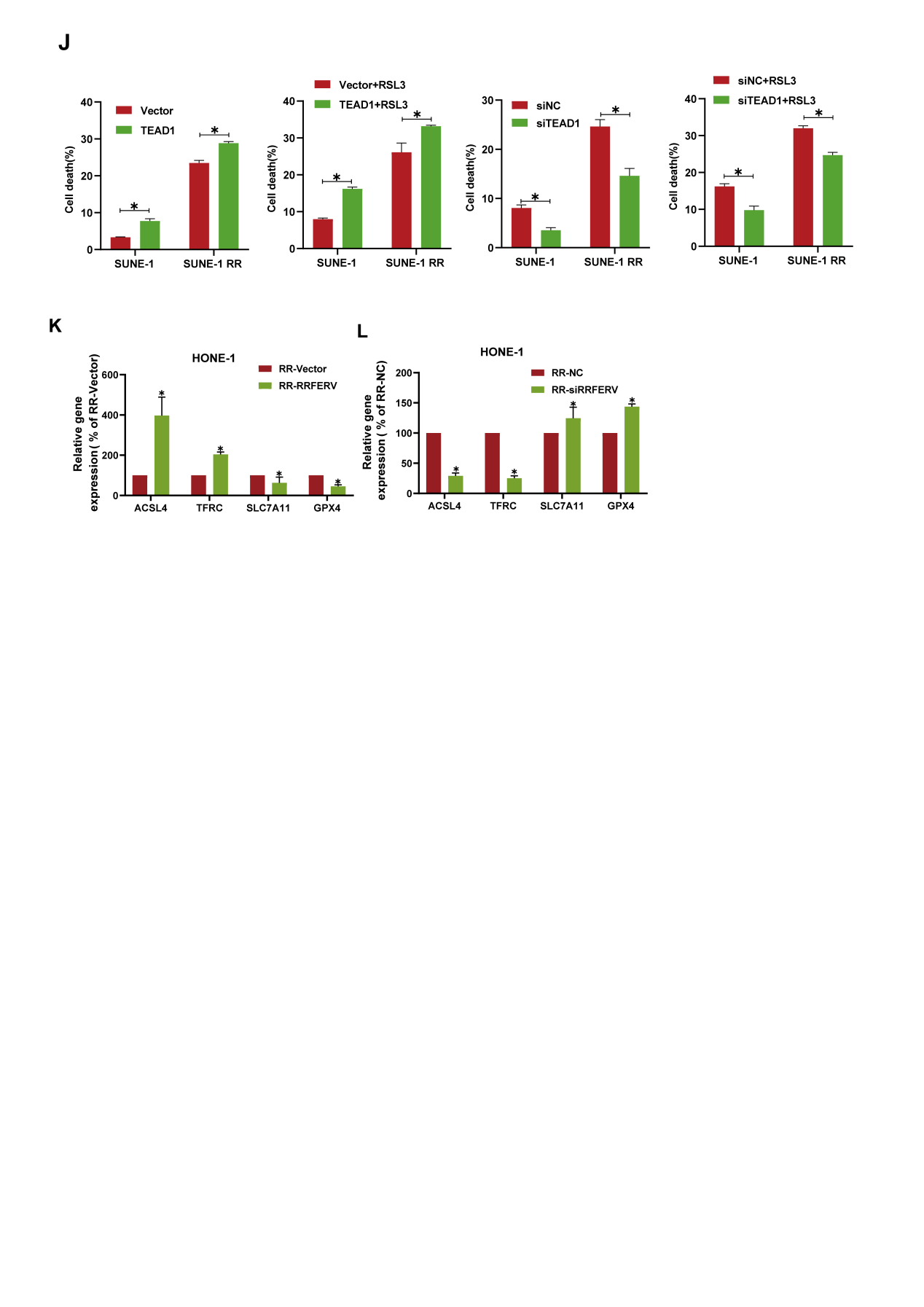
**

**Supplement Figure 6. legends**

1. RNA-seq datasets from paired radiotherapy-tolerant cell lines (SUNEERR and SUNE) were analyzed on 18 pairs of nasopharyngeal carcinoma and normal tissue samples. **(B)** Colony formation assay detect the radiation-tolerant cell lines (HONERR and HONE, SUNERR and SUNE). **(C-D)** Detection of the expression levels of ferroptosis-related signal pathway members using qPCR in NPC RT sensitive and tolerant cell lines. **(E)** Lipid peroxidationin was detected to reveal ferroptosis in RR and wild type cell lines with indicated concentration RSL3 or Erastin. **(F)** Lipid peroxidation was detected in RR and wild-type cell lines transfected with RRFERV or vector control with the indicated concentrations of RSL3 or Erastin to reveal ferroptosis. **(G-H)** Flow experiment was detected in cells upon RRFERV overexpression or knockdown with and without RSL3. **(I-J)** Flow experiment was detected in cells upon TEAD1 overexpression or knockdown with and without RSL3. **(K)** QPCR was detected genes associated with ferroptosis in cells upon RRFERV overexpression or knockdown in HONE cells.
